# Photouncaged Sequence-specific Interstrand DNA Cross-Linking with Photolabile 4-oxo-enal-modified Oligonucleotides

**DOI:** 10.1038/srep10473

**Published:** 2015-05-28

**Authors:** Jingjing Sun, Xinjing Tang

**Affiliations:** 1 State Key Laboratory of Natural and Biomimetic Drugs, the School of Pharmaceutical Sciences, Peking University, 38 Xueyuan Road, Beijing 100191, China

## Abstract

DNA cross-linking technology is an attractive tool for the detection, regulation, and manipulation of genes. In this study, a series of photolabile 4-oxo-enal-modified oligonucleotides functionalized with photosensitive ο-nitrobenzyl derivatives were rationally designed as a new kind of photocaged cross-linking agents. A comprehensive evaluation of cross-linking reactions for different nucleobases in complementary strands under different conditions suggested that the modified DNA oligonucleotides tended to form interstrand cross-linking to nucleobases with the potential of thymidine > guanosine » cytidine ~ adenosine. Different from previous literature reports that cytidine and adenosine were preferential cross-linked nucleobases with 4-oxo-enal moieties, our study represents the first example of DNA cross-linking for T and G selectivity using 4-oxo-enal moiety. The cross-linked adducts were identified and their cross-linking mechanism was also illustrated. This greatly expands the applications of 4-oxo-enal derivatives in the studies of DNA damage and RNA structure

DNA interstrand cross-linking widely exists in biological systems and is a useful chemical biology tool as well as an effective strategy for cancer therapy. It is of potential biological and clinical interests to investigate functional moieties in oligonucleotides forming covalent bonds with specific nucleotides in a sequence-specific manner under physiological conditions^1^,^2^. Such DNA interstrand cross-linking approaches have been reported in gene expression inhibition^3^, DNA damage mimicking^4^, and RNA structure investigation^5^. Indeed, extremely low quantities of defined interstrand cross-link (ICL) adducts could be isolated from natural sources for further ICL mechanistic studies. If DNA was treated by external cross-linking reagents, only small amount of desired ICLs could be obtained with large amount of complex mixtures of mono-, and inter- and intrastrand cross-linked adducts. Therefore, considerable research efforts have been focusing on efficient and highly sequence-specific cross-linked oligonucleotides.

Constructing oligonucleotides with a functional moiety that is reactive toward nucleotides of their complementary strands have been achieved recently, including halocarbonyl^6^,^7^, aziridine^8^,^9^,^10^ or other strained rings^11^,^12^,^13^. These functional moieties, however, typically lack sufficient stability, resulting in low yields and/or off-target effects. More promising results are achieved when a reactive cross-linking moiety is produced from a stable precursor, e.g. disulfide bonds^3^, quinone methides^6^,^11^,^14^, carbazoles^5^,^15^,^16^, benzophenone derivatives^17^, phenylselenyl derivatives of pyrimidines^18^, abasic site^19^,^20^, and furan derivatives^13^,^21^,^22^,^23^. Among them, Madder reported that methylene blue-induced ^1^O_2_ formation triggered furan oxidation for further DNA cross-linking^13^,^21^,^22^. And Sasaki *et al* described an alternative cross-linking strategy through oxidative deprotection of reactive vinyl groups^24^,^25^,^26^,^27^,^28^,^29^,^30^. However, all of these approaches required extra chemical and/or biological reagents (such as F^-^, H_2_O_2_, enzyme)^6^,^31^,^32^ which may limit their biological applications. It will be beneficial to apply extra clean triggers, such as light activation, to realize concise activation in spatiotemporal resolution. In this respect, photolabile ODNs are of particular interest for artificial manipulation of gene expression, as light has stood out as an excellent non-invasive trigger due to high spatiotemporal resolutions^33^,^34^,^35^,^36^. Psoralen analogues have been used as T-selective photo-cross-linkers, however, this photo-cross-linking reaction requires a TpA step of the target DNA sequence. In addition, the photo-cross-linked ODNs was produced via a [2+2] cycloaddition between psoralen and thymine^37^,^38^. Investigation of light-controlled reversible DNA photoligation was also achieved by Fjimoto via cyanovinyl carbazole nucleosides in a [2+2] cycloaddition manner at the presence of template ODNs^39^,^40^.

The 4-oxo-enal moieties generated through oxidation of furan ring have been previously reported^13^,^21^,^22^,^23^ for ICL formation between the exocyclic amine of adenine or cytosine and the aldehyde group of 4-oxo-enal moiety^13^,^21^,^22^,^23^. Different from previous 4-oxo-enal moieties that contain two carbonyl groups, we developed a 4-oxo-enal moiety with one carbonyl and one amide groups. In this study, we synthesized photo-cross-linking ODNs containing caged 4-oxo-enal moieties at the end of the ODNs that can be uncaged with light activation. These photolabile ICL precursors remained stable during and after their synthetic incorporation into duplex DNA until the ketals were deprotected in neutral conditions by irradiation with light, resulting in the formation of an enal species. The *in situ* generated species showed high reactivity towards cross-linking with the nucleobases in the complementary strand, as shown in Fig. 1. Herein, we described in detail the synthesis of ODN**1**, ODN**2** and ODN**3**, evaluation of their cross-linking reactivity and selectivity for thymidine and guanosine, and analysis of the cross-linked adducts. And we also proposed a cross-linking mechanism between 4-oxo-enal and the corresponding thymidine.

## Results and Discussion

### Synthesis and characterization of photolabile ODNs

We synthesized two kinds of 4-oxo-enal derivatives that were caged with two photolabile *ο*-nitrobenzyl moieties and they were incorporated into ODNs (Fig. 2). Firstly, (E)-ethyl 4-oxo-but-2-enoate was protected with two *ο*-nitrobenzyl derivatives by treating with *p*-toluenesulfonic acid in toluene, respectively. The subsequent hydrolysis of ester afforded the carboxyl products (**3** and **4**) under alkaline condition. The carboxylic acids were then activated with DCC and *N*-hydroxysuccinimide for further coupling with the terminal amine of ODNs. Different from previous 4-oxo-enal derivatives with ketone at 4^th^ position, we presented 4-oxo-enal derivatives with amide at 4^th^ position. The coupling of oligonucleotides with 4-oxo-enal derivatives was carried out in 0.1 M NaHCO_3_ solution at 50 °C for 10 h and the desired ODN products were obtained over 90% yields based on HPLC analysis (Figure S1 for ODN**1**) and confirmed by ESI mass spectra.

Photoinduced release of terminal labelled ODNs was examined by reversed-phase HPLC. Under UV irradiation at 365 nm (7 mW/cm^2^), the peak of photolabile ODNs (1 μM) disappeared gradually, while a new peak at 9.8 min on HPLC traces simultaneously appeared, which was confirmed as uncaged ODN by ESI mass spectrum (Figure S2). Kinetic study of the photocleavage reaction revealed that 5 min light irradiation mainly produced the target uncaged ODN. Elongating the irradiation time, the main product peak decreased, accompanied by the increase of by-product peak. After 5 min UV irradiation, the freshly purified deprotected ODN**1** was incubated alone for 24 hours at 37 ^o^C. The same by-product that was probably the self-cross-linked product with the same molecular weight was found and validated by HPLC, PAGE gel and MS (Figure S3 and S4). Therefore, 5 min UV irradiation was chosen for all the following cross-linking studies.

### Evaluation of interstrand cross-link formation and nucleobase selectivity

**Figure 1:**
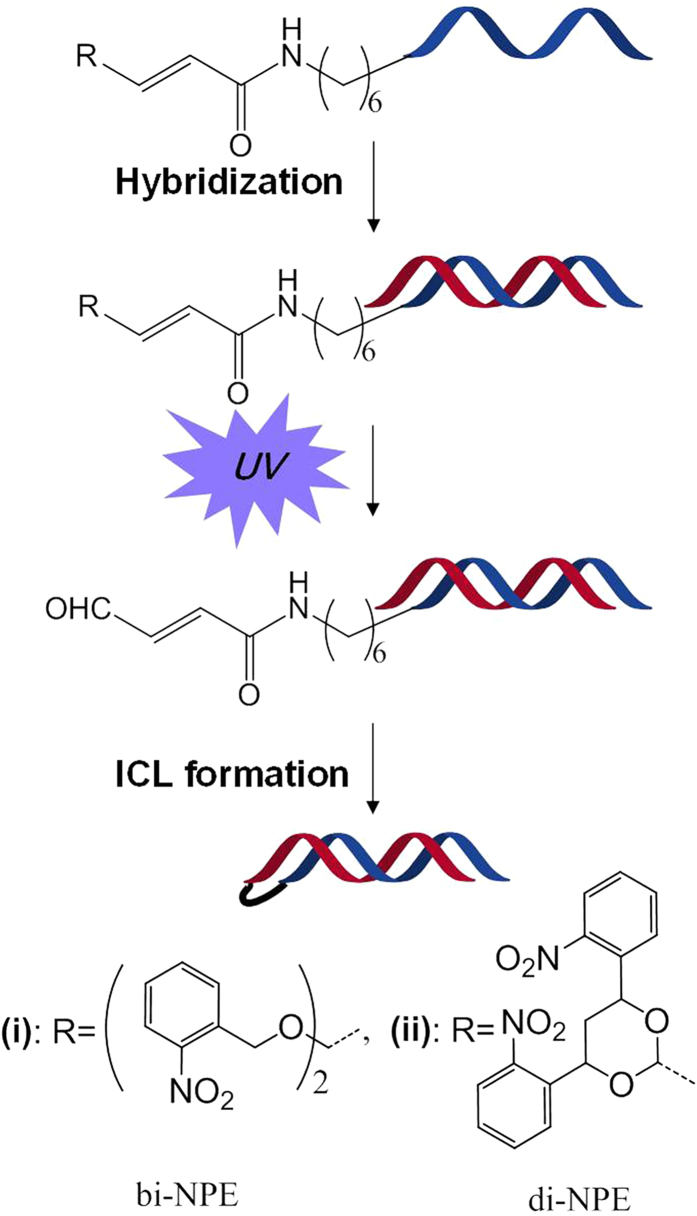
Interstrand cross-link formation of 4-oxo-enal functionalized ODNs triggered by UV light.

**Figure 2:**
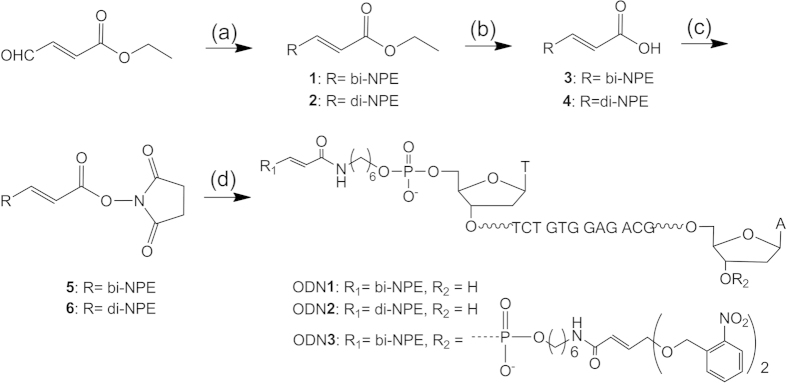
Synthesis of photolabile ODNs (ODN1, ODN2, and ODN3). (**a**) compound **1**: o-nitrobenzyl alcohol*, p*-toluenesulfonic acid, toluene, molecular sieves, 54.7%, compound **2**: 1,3-bis(2-nitrophenyl)propane-1,3-diol, *p*-toluenesulfonic acid, toluene, molecular sieves, 84.4%; (**b**) NaOH, THF, 95.6% for **3**, 92.4% for **4**; (**c**) NHS, DCC, THF, 60.1% for **5**, 86.2% for **6**; (**d**) 0.1 M NaHCO_3_, pH = 8.4.

**Figure 3:**
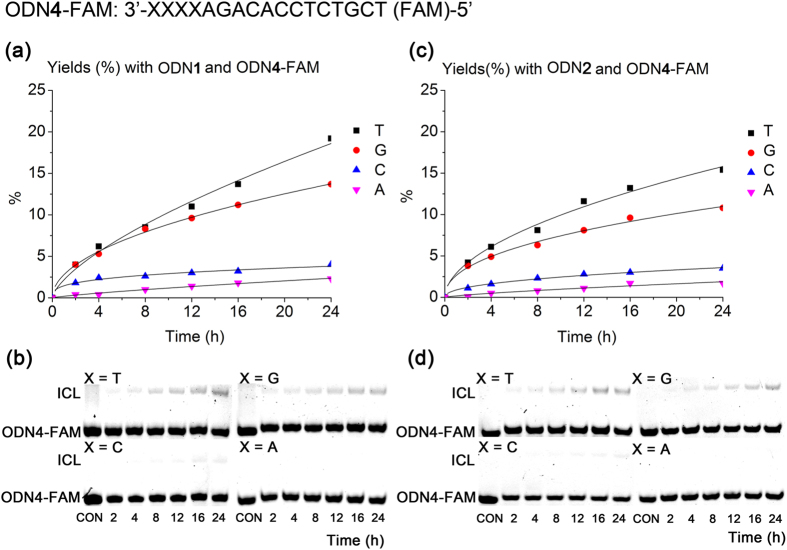
Interstrand cross-linking reactions of two duplexes ODN1/ODN4 and ODN2/ODN4. Cross-linking conditions: [ODN1 or ODN2] = 10 μM, ODN4 = 1 μM, 10 mM PBS buffer, 100 mM NaCl, 37 °C, pH 7.0. The reaction was followed by electrophoresis using 20% denatured polyacrylamide gel. The fast- and slow-moving bands represent ODN4 and the cross-linked products, respectively. The cross-linking yields were obtained by quantification of the bands by FAM fluorescence, and they were then plotted against time. (**a**) Yields (%) obtained with ODN1 and ODN4-FAM (X = T, G, C, A). (**b**) Gel-shift analysis of the reaction with ODN1. (**c**) Yields (%) obtained with ODN2 and ODN4-FAM (X = T, G, C, A). (**d**) Gel-shift analysis of the reaction with ODN2. CON =control.

**Figure 4:**
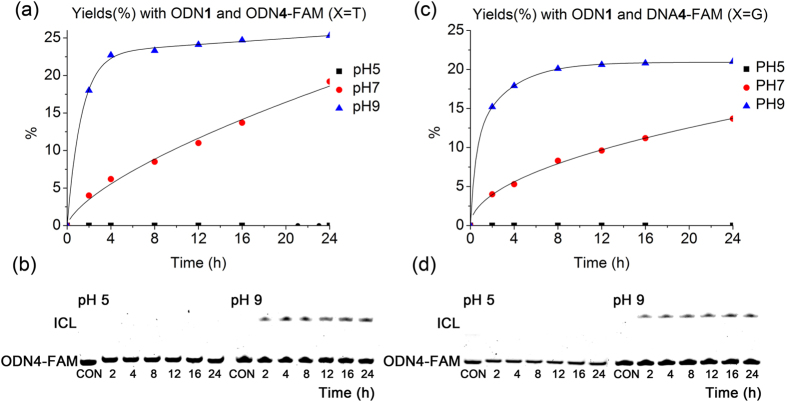
Cross-linking reaction with ODN**1** and ODN**4** (X = T, G). Cross-linking conditions: ODN**1** = 10 μM, ODN**4** = 1 μM, 10 mM MES buffer, 100 mM NaCl, 37 °C, pH 5.0; 10 mM carbonate buffer, pH 9.0. The reaction was followed by electrophoresis using 20% denatured polyacrylamide gel. The fast- and slow-moving bands represent ODN**4** and the cross-linked products, respectively. The cross-linking yields were obtained by quantification of the bands by FAM fluorescence, and they were then plotted against time. (**a**) Yields (%) obtained with ODN**1** and ODN**4** (X = T). (**b**) Gel-shift analysis of the reaction with ODN**4** (X = G). (**c**) Yields (%) obtained with ODN**1** and ODN**4** (X = T). (**d**) Gel-shift analysis of the reaction with ODN**4** (X = G). CON=control.

The ODNs (ODN**1** or ODN**2**) contained diffirent photolabile cross-linkers at 5′ terminus were used for the comparison of interstrand cross-linking efficiency. The sequence (TCTGTGGAGACGA) of target DNA strands is the antisense sequence of JAK2 gene, which is associated with chronic myeloproliferative disease (CMPDs). The complementary target ODN**4** (5′-FAM-TCGTCTCCACAGAXXXX, X = G, A, C, T, 6-carboxyfluorescein (6-FAM) labelled at the 5′ end) with four different nucleobases was also designed to evaluated the selectivity of the cross-linking nucleobase. Without light activation, both photolabile ODNs (ODN**1** and ODN**2**) showed no cross-linking activity to its complementary ODN**4** (Figure S5). However, once the duplexes of photolabile ODNs and their complementary target sequences were photoactivated and incubated under neutral conditions at 37 °C, we observed the formation of a band with slower migration in 20% denaturing PAGE gel, indicative of a cross-linked adduct that was later confirmed as ICL oligonucleotide products by ESI-MS (Figure S11). And the yield of ICL formation could be quantified through characterization of FAM fluorescence of two bands with the lower and higher mobility. The reactivity of the uncaged ODNs (**1** and **2**) toward the target ODN**4** with different nucleobases (X =T, G, C, A) were illustrated in Fig. 3. Different from literature reports that adenosine and cytidine were most reactive nucleobases against 4-oxo-enal^21^,^22^,^23^, ODN**4** (X **=** T) actually demonstrated the most reactive and efficient cross-linking with uncaged ODN**1** (Figs. 3a and 1b). And their cross-linking activities decreased in the order of thymidine > guanosine » cytidine ~ adenosine. This also explained that the possible self-cross-linked by-product was easily happened due to the last thymidine at 5’ end of antisense sequence close to 5’-end labeled flexible 4-oxo-enal moiety. Similar to ODN**1**, uncaged ODN**2** maintained the same selectivity for T and the same order of cross-linking activity toward different nucleobases with only a little slower reaction rate (Figs. 3c and 1d). To gain more insight of the cross-linking between 4-oxo-enal and nucleobases, the activities of the ODNs (**1** and **2**) toward the complementary target ODN**4** (X = T, G) were further carried out under acid and basic conditions as shown in Fig. 4 and Figure S6. It turned out that the interstrand cross-linking in buffer at pH 9 underwent much more efficiently than pH 7 for either ODN**1** or ODN**2**. At the same time, no crosslinked adducts were visible in buffer at pH 5. While literature-reported DNA cross-linking between 4-oxo-enal moiety and cytidine or adenosine are preferentially under low pH, such as pH 5. This pH dependence of cross-linking suggested that amide group of thymidine or guanosine might be deprotonized and nitrogen of amide group might couple with 4-oxo-enal group through Michael addition under basic condition, while no cross-linking was observed under acidic condition. This observation was similar to a recent report with a reactive vinyl modified thymidine for interstrand cross-linking^26^. Further temperature dependence of cross-linking reaction indicated that high temperature accelerate the interstrand cross-linking for both photolabile ODNs (**1** and **2**) with their complementary target sequence ODN**4** (X =T and G) (Figure S7 for ODN**1** and S8 for ODN**2**).

**Figure 5:**
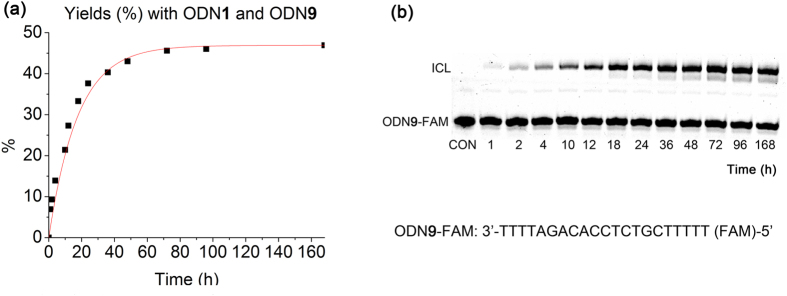
Cross-linking reaction with ODN**3** and ODN**9**. Cross-linking conditions: ODN**3** = 10 μM, ODN**9** = 1 μM, 10 mM PBS buffer, 100 mM NaCl, 37 °C, pH 7.0. The reaction was followed by electrophoresis using 20% denatured polyacrylamide gel. The fast- and slow-moving bands represent ODN**4** and the cross-linked products, respectively. The cross-link yield was obtained by quantification of the bands by FAM fluorescence, and it was plotted against time. (**a**) Yields (%) obtained with ODN**3** and ODN**9**. (**b**) Gel-shift analysis of the reaction with ODN**3**. CON =control.

We then further designed and synthesized ODN**3** with two photolabile 4-oxo-enal at both 5′ and 3′ ends of the same ODN sequence to evaluate how two different terminal labeling influoence DNA cross-linking and how far 4-oxo-enal moiety could reach the thymidine nucleobase for interstrand cross-linking. Upon light activation, the released 4-oxo-enal moieties at both ends of the oligonucleotide could cross-link to the complementary oligonucleotides (**5**, **6**, **7** and **8**) with cross-linking yield up to 25.8% (Figure S9). By placing the thymidine at different position of one terminal dangling sequence, no obvious difference of cross-linking ability was observed, suggesting that the flexible linker was long enough to reach the thymidine as far as 5th nucleobase away. We then further examined cross-link formation between ODN**3** and ODN**9** which contained ploy T at both 5′ and 3′ ends of the ODN sequence. After light activation and further incubation in PBS buffer (10 mM, pH 7.0, 100 mM NaCl) at 37 °C for 168 hours, the gel evaluation and analysis of ODN samples at different time points indicated that up to 46.9% of cross-linked adducts of uncaged ODN**3** and ODN**9** was observed after 60 hours incubation, as indicated in Fig. 5.

### Determination of the cross-linked adducts

**Figure 6:**
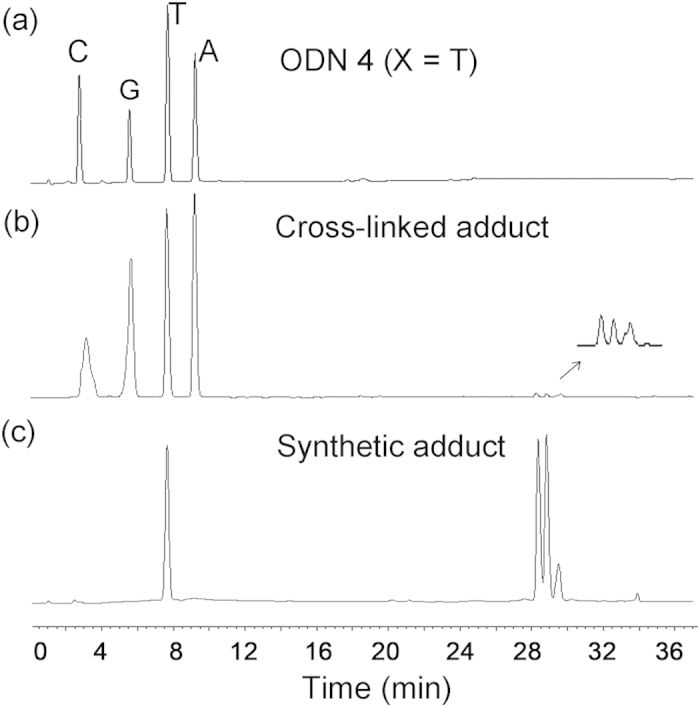
(**a**) HPLC analysis of the enzymatic hydrolyzates of unmodified oligodexynucleotides ODN**4** (X = T) for negative control experiment. (**b**) HPLC analysis of the enzymatic hydrolyzates of the cross-linked products obtained at pH 9.0. (**c**) HPLC analysis of the peaks appearing at 28~30 min of chemically synthesized cross-linked adducts that were identical to the enzymatically digested MS cross-linked adducts.

Under our situation, the photocaged 4-oxo-enal showed almost no reactivity to A and C, suggesting that no addition of 4-oxo-enal with exocyclic nitrogen atom was observed. To further characterize the cross-linked adducts, we isolated cross-linked adducts between uncaged ODN**1** and ODN**4** (X =T) through PAGE under denaturing conditions. The purified adduct was further confirmed by HPLC (Figure S10) and MS analysis (Figure S11, 12). The results indicated the stable adduct had the observed molecular weight of the sum of uncaged ODN**1** and ODN**4** (X =T) ([M-H]^-^ calcd: 9387.3, found: 9389.1), while mainly loss of a water molecule of ODN**1** and ODN**4** was observed (X =G) ([M-H]^-^ calcd: 9470.3, found: 9469.9). For an accurate characterization of formed adducts and identification of cross-linking, further enzymatic digestion of the purified cross-linked oligonucleotide between ODN**1** and ODN**4** (X = T) by Snake venom phosphodiesterase (SVPDE) and Alkaline phosphatase (ALP) was carried out to confirm the nucleobase-specific formation. Figs. 6a,b shows RP-HPLC traces obtained after enzymatic digestion of native ODN duplex and cross-linked ODN (The HPLC analysis of the enzymatic hydrolyzates of the cross-linked products obtained at pH 7.0 was shown in Figure S13, showing no obvious difference with the data from pH 9.0). The four unmodified C, G, T and A can be clearly identified. There were three new tiny peaks appearing during 28 to 30 min. Each of these three fractions was collected and characterized by ESI-MS, respectively. MS results indicated that all three fractions have the same molecular weights and further high resolution MS showed that these species are adducts directly formed by thymidine and the 4-oxo-enal derivative ([M+Cl]^-^, calcd: 476.18051, found: 476.18052; [M+HCOO]^-^, calcd: 486.20932, found: 486.20836). The enzymatic hydrolyzates results of cross-links of ODN**1** and ODN**4** (X = G) is also shown in Figure S14, the adducts appeared in nearby 19 min, which is validated by ESI-MS ([M+H]^+^, calcd: 448.35)

To investigate whether cross-links caused by the photoinduced generation of free radical from broken C-O bond of acetal, we added 2,2,6,6-Tetramethyl-1-piperidinyloxy (TEMPO), a kind of radical inhibitor, to the cross-linking reaction solution (Figure S15). From the Gel analysis, no distinct difference can be observed with or without TEMPO. So the cross-linking mechanism is not related with free radical. According to literature reports^26^, a general mechanism involves the initial cross-linking of 4-oxo-enal moiety with the exocyclic nitrogen atom of nucleobases of adenosine and cytidine (N6 of A and N4 of C), followed by the addition of adjacent endocyclic nitrogen atom of the double bond to form a pentacycle, which can be more easily achieved under acidic condition. In this case, no A and C cross-linked adducts were observed no matter under acidic, neutral or basic conditions (Figure S16), while T has the highest activity to uncaged 4-oxo-enal moiety. Since T has been previously reported^26^ that N3 has potential to nucleophilic potential to nucleophilic attack to halogenated molecules or α, β double bonds under basic condition due to deprotonization of amide moiety of T. Further pH dependency of reactivity combined with high selectivity of T suggested that the deprotenized nitrogen of amide attacked the 4-oxo-enal moiety to form cross-linked adducts, most likely through Michael addition to double bond of α, β unsaturated aldehyde by N atom of thymidine amide.

**Figure 7:**
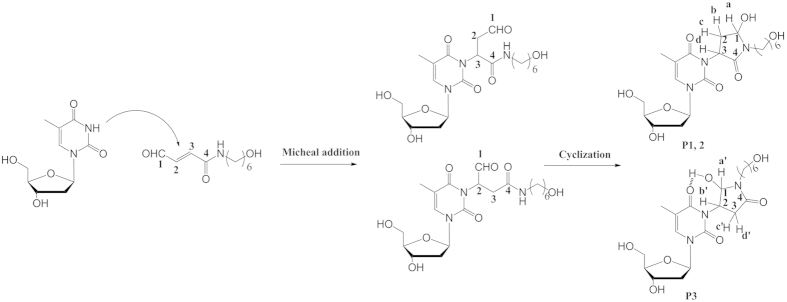
The proposed mechanism and structure of the cross-linked adducts.

**Figure 8:**
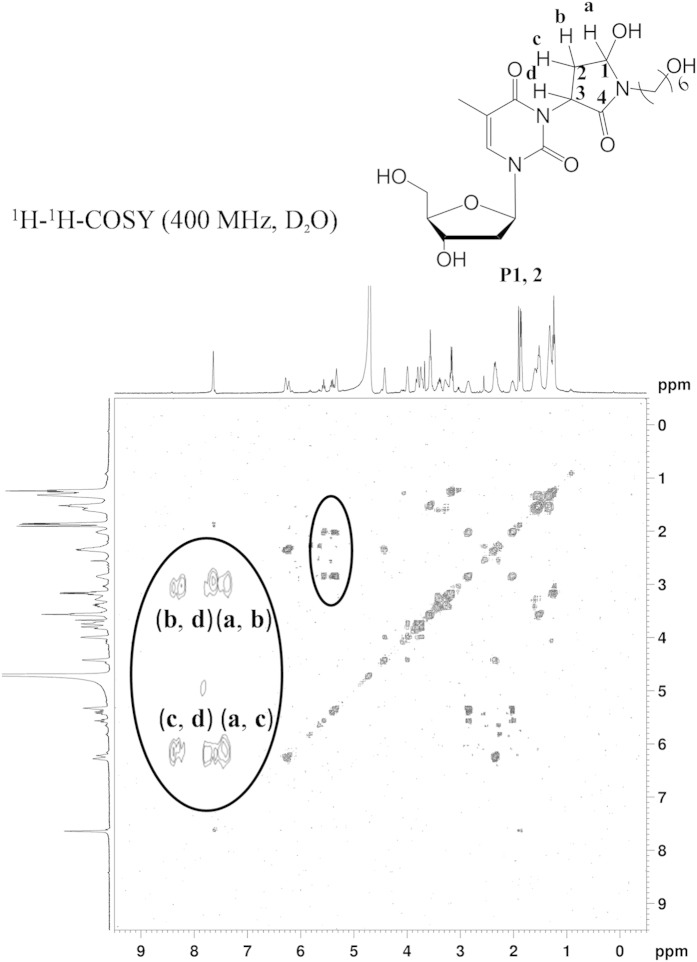
^1^H-^1^H COSY of the cross-linked adducts (P1 and 2).

In order to unambiguously identify the structure of the cross-links, a α, β unsaturated aldehyde analogue (**11**) was synthesized as cross-linker and then reacted with thymidine in PBS buffer (10 mM). The reaction mixture was first roughly purified by silica gel column to remove most unreacted thymidine. Further purification of obtained residue was performed with HPLC, as showed in Fig. 6c. Three new peaks were observed during 28 to 30 min which corresponded to the same three product peaks of enzymatically digested cross-linked adducts. These three fractions were collected respectively and analyzed by ESI-MS. MS results indicated that all three fractions have the same molecular weights as those of the enzymatic digestion fractions. HPLC and MS results indicated that the chemical synthetic products should have the same structures with DNA cross-linked adducts. Furthermore, all those chemical synthetic products were characterized through ^1^H NMR, ^13^C NMR, ^1^H-^1^H COSY. After analyses of all above NMR data and MS of isolated adducts, a mechanism and adducts’ structures were proposed, as illuminated in Fig. 7. The vanishing proton signal of double bond demonstrated that N3 atom of thymidine was deprotonized and coupled to double bond of α, β unsaturated carbonxyl group through nucleophilic attack. Beyond our expectations, an intramolecular cyclization reaction happened between the NH of amide (C4) and aldehyde (C1) groups, as evidenced by the disappearance of proton signal of aldehyde and NH of amide (C4-NH). Fractions of P1 and P2 (Fig. 6b) with the equal intensity in HPLC trace have the same NMR data, which indicates that they are stereoisomers. Their structures with Michael addition at C3 position were confirmed by ^1^H-^1^H COSY with two couplings of Ha (5.33 ppm, 1H) and Hb,c (2.03-2.01 ppm, 2.85 ppm, 2H), Hb,c (2.03-2.01 ppm, 2.85 ppm, 2H), and Hd (5.59 – 5.38 ppm, 1H) as shown in Fig. 7 (P1 ,P2) and Fig. 8. The difference with Peak 3 comes from Michael addition to different carbon (C2, Fig. 7) of double bond of α, β unsaturated aldehyde to form P3 that mainly reflects in ^1^H-^1^H COSY (Figure S17 for Peak 3) with two coupling of Ha’ (5.48 ppm, 1H) and Hc’ (2.60 ppm, 1H), Hd’ (2.35-2.29 ppm, 1H) and Hb’ (5.78 ppm, 1H). In the structure of P3, intramolecular H-bond formation can be easily performed between 4^th^ carbonyl of thymine and the hydroxyl group generated from the addition reaction of NH of amide and aldehyde group, which contributes to lower polarity and longer retention time in RP-HPLC analysis of P3. Under the same conditions, the cross-linking of guanosine and **11** was not achieved. Instead, cyclization of **11** was isolated. This is probably due to lower nucleophilic property of N of guanosine than thymidine.

## Conclusions

A simple and versatile strategy for photocaged ODN interstrand cross-linking was developed for nucleic acids. Photolabile 4-oxo-enal derivatives were rationally designed and synthesized for terminal labeling of ODNs. The labeled ODNs cross-linked to their complementary oligonucleotides upon light activation of 4-oxo-enal moiety. Further investigation indicated the highly selective cross-linking for thymidine and guanosine instead of preferential cross-linking nucleotides (A, C) reported in literature. And basic condition actually promoted the formation of cross-linked adducts, suggesting that these adducts may be formed through nucleophilic attack of thymidine to 4-oxo-enal moiety. By analysis of cross-linked adducts after enzyme digestion and chemically synthesized adducts, the same three possible adducts with the same molecular weights were generated for both situations. With the assistance of ^1^H NMR, ^13^C NMR, ^1^H-^1^H COSY and as well as mass spectra of isolated adducts, the structures of cross-linked adducts were identified and their detailed cross-linking mechanism was proposed. Our results provide, to the best of our knowledge, the first ODN cross-linking example of 4-oxo-enal derivatives for T and G selectivity instead of A and C, which will greatly expand applications of 4-oxo-enal derivatives in the studies of DNA damage and RNA structure.

## Methods

### Synthesis, purification and characterization of caged oligonucleotides

A DMF solution of **5** (1.0 mg) in equivalent volume was added to a solution of the commercially available oligonucleotides modified by 5′ terminal amine at 0.1 mM concentration in 0.1 M NaHCO_3_ aqueous solution. Samples were shaken in a Thermo shaker at 1000 rpm and 50 °C for 10 h. The mixture was extracted with DCM to remove unreacted **5**. The supernatant was collected and concentrated. The oligonucleotides were then precipitated using 3 M NaOAc. The caged oligonucleotides were further purified through reverse-phase HPLC with running conditions: Phase A, 0.05 mM TEAB; Phase B, MeCN. The following step-wise gradient was used with flow rate of 1.0 mL /min, 0-20% MeCN in 20 min. The column temperature was maintained at 40 °C. The product fraction was collected and further characterized with ESI-MS. ODN**1**: [M-H] ^-^ calcd: 4564.2, found: 4565.0; ODN**2**: [M-H] ^-^ calcd: 4576.2, found: 4576.6; ODN**3**: [M-H] ^-^ calcd: 5113.7, found: 5114.3.

The solution of photoliable ODNs (**1** and **2**, 1 μM) were irradiated with a UV-LED lamp (365 nm, 7 mW/cm^2^, Shenzhen Lanpulike Biotechnology Co. Ltd.) for 5 min to test the uncaging of photocleavable groups. The irradiated oligonucleotides (ODNs **1** and **2**) were further confirmed to be the uncaged product by ESI-MS [M-H] ^-^ calcd: 4275.9, found: 4277.8.

### Cross-linking reaction

The reactions between caged oligonucleotides and complementary strands were described as the following general procedure. A mixture of FAM-labeled ODN**4** (1 μM) and ODN**1** (10 μM) in a buffer solution containing 100 mM NaCl was annealing from 95 °C to room temperature. The samples were then irradiated with a UV-LED lamp (365 nm, 7 mW/cm^2^, Shenzhen Lanpulike Biotechnology Co. Ltd.) for 5 min. After irradiation, the mixture was incubated in buffer (10 mM). The cross-linking reactions at pH 5.0, 7.0 and pH 9.0 were performed in MES buffer, PBS buffer and carbonate buffer. Aliquots (5 μL) were taken at regular intervals to analyze the cross-linked products.

### Gel shift assays

A 20% polyacrylamide gel (acrylamide: bisacrylamide = 19:1) with 1×Tris-Borate-EDTA (TBE) buffer (pH 8.2) and 7 M urea was used for all analyses. Samples mixed with formamide loading buffer were heated at 90 °C for 3 min and placed in ice bath before being loaded into gel. Gels were run for 2.5 h at a voltage of 120 V. Gels were imaged with Molecular Imager ChemiDoc™ XRS+. The fluorescence intensities of bands on each gel were integrated by Image Lab™ version 2.0 with automated lane and band finding using a local method background correction for each lane.

### Enzymatic digestion

The purified cross-linked duplex (1 nmol) was dissolved in aqueous solution (100 μl) with Tris-HCl buffer pH 8.0 (10 μL, 500 mM) and MgCl_2_ (10 μL, 100 mM). Snake venom phosphodiesterase (SVPDE, 10 μl, 0.11 U/μL) was added to the reaction mixture and the mixture was then incubated overnight at 37 °C. Alkaline phosphatase (ALP, 5 μl, 1 U/μL, from bovine intestinal mucosa, Sigma Aldrich) was then added and the reaction mixture was further incubated at 37 °C for 1 h. The mixture was directly injected into RP-HPLC to collect all nucleotide fractions at 260 nm absorbance. The running conditions: Phase A, 0.05 mM TEAA; Phase B, MeCN. The following step-wise gradient was used with flow rate of 1 mL/min, 0-10% MeCN in 30 min, 10%-35% MeCN in 30 min. The column temperature was maintained at 40 °C. All collected peaks were analyzed by ESI-MS.

## Additional Information

**How to cite this article**: Sun, J. and Tang, X. Photouncaged Sequence-specific Interstrand DNA Cross-Linking with Photolabile 4-oxo-enal-modified Oligonucleotides. *Sci. Rep.* **5**, 10473; doi: 10.1038/srep10473 (2015).

## Supplementary Material

Supplementary Information
