# Supplementary material for: Photouncaged Sequence-specific Interstrand DNA Cross-Linking with Photolabile 4-oxo-enal-modified Oligonucleotides
Source: Sci Rep. 2015 May 28;5:10473. doi: 10.1038/srep10473 (PMC4650688; doi:10.1038/srep10473)

Supporting information

Photouncaged sequence-specific Interstrand DNA Cross-Linking with photolabile 4-oxo-enal-modified Oligonucleotides

Jingjing Sun, Xinjing Tang*

State Key Laboratory of Natural and Biomimetic Drugs, the School of Pharmaceutical Sciences, Peking University, Beijing 100191, China

* Tel: +86-010-82805635; Fax: +86-010-82805635; Email: xinjingt@bjmu.edu.cn

MATERIAL AND METHODS

General methods

All Chemical reagents were purchased from Alfa Aesar, Sigma Aldrich, or J&K, and used without further purification. All oligonucleotides were purchased from Sangon Biotech (Shanghai) Co. Ltd with HPLC purification. All organic reactions were monitored by TLC using commercial Merck-Plates coated with silica gel GF_{254} (0.24 mm thick). Flash column chromatography was performed with silica gel purchased from Qingdao Haiyang Chemical Company (200-300 mesh). The spectra of ^{1}H NMR (400 MHz), ^{13}C NMR (101 MHz) and ^{1}H-^{1}H COSY were recorded on a Bruker spectrometer (Bruker 400MHZ Advance Ⅲ) at 25 °C and the NMR data were processed using Bruker TOPSPIN 3.0. MS were measured on Q-TOF spectrometer using electrospray ionization (ESI). HPLC were performed with Alliance e2695 and Agilent XDB C-18 column (50 x 4.6 mm, 1.8 μm beads). The concentrations of all oligodeoxynucleotides were measured in 2000/2000c Spectrophotometer Nucleic Acid/ Protein Analyzer. All photoirradiation experiments with ODN samples were carried out with UV lamp (365 nm, 7 mW/cm^2). Gels were imaged with Molecular Imager ChemiDoc™ XRS$^+$. The fluorescence intensities of bands on each gel were integrated by Image Lab^{TM} version 2.0 with automated lane and band finding using a local method background correction for each lane. When necessary, the operation was conducted in a dark room.

Synthesis of photolabile 4-oxo-enal derivatives

*(E)-ethyl 4,4-bis((2-nitrobenzyl)oxy) but-2-enoate (**1**).*

To the dry toluene solution (15 mL) of (E)-ethyl 4-oxobut-2-enoate (267 μL, 2 mmol, 1.0 equiv.) and 2′-nitrobenzyl alcohol (689 mg, 4.5 mmol, 2.25 equiv.), a catalytic amount of *p*-toluenesulfonic acid (56 mg, 0.2 mmol, 0.1 equiv.) was then added. And the mixture was stirred with molecular sieves at 45 °C overnight. The solvent was removed via rotary evaporation under reduced pressure. Then the residue was diluted with ethyl acetate and washed with sodium bicarbonate. The organic layer was then dried over anhydrous Na_2SO_4. After removal of solvents, further purification by silica gel column chromatography (PE:EA=5:2) yielded 455 mg (54.7%) of **1** as a white solid. ^1H NMR (400 MHz, $CDCl_3$) δ 8.06 (d, *J* = 8.1 Hz, 2H), 7.80 (d, *J* = 7.7 Hz, 2H), 7.65 (t, *J* = 7.6 Hz, 2H), 7.44 (t, *J* = 7.7 Hz, 2H), 6.90 (dd, *J* = 15.9, 3.8 Hz, 1H), 6.29 (d, *J* = 15.9 Hz, 1H), 5.47 (d, *J* = 2.8 Hz, 1H), 5.02 (q, *J* = 14.8 Hz, 4H), 4.23 (q, *J* = 7.1 Hz, 2H), 1.31 (t, *J* = 7.1 Hz, 3H). ^{13}C NMR (101 MHz, $CDCl_3$) δ 165.79, 147.40, 141.74, 134.22, 133.99, 128.96, 128.45, 125.68, 124.97, 99.74, 64.75, 61.09, 14.39. ESI-MS: $[M+Na]^+$ cacld: 439.12, found: 439.44.

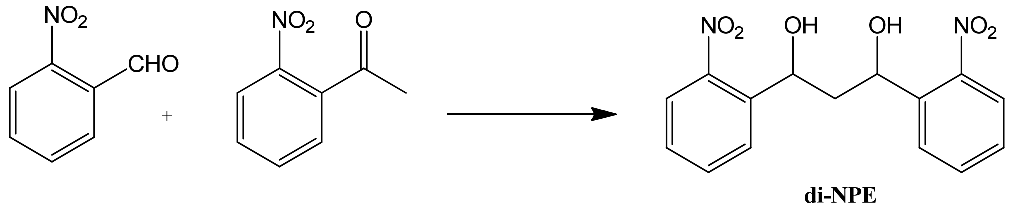

Scheme S1. The synthesis of **di-NPE**.

*1, 3-di (2-nitrophenyl) propane-1, 3-diol (**di-NPE**).*

The systhesis of ***di-NPE*** was according to literature procedure with minor modification. To a solution of 2-nitrobenzaldehyde (757 mg, 5.0 mmol, 1.0 equiv.) and 2′-nitroacetophenone (908 mg, 5.5 mmol, 1.1 equiv.) in 35 mL of CH_3CN/H_2O (4:1) was added K_2CO_3 (93 mg, 0.7 mmol, 0.13 equiv.). The mixture was stirred at 0 °C overnight. The organic solvent was evaporated under reduced pressure and the

aqueous residue was extracted with ethyl acetate. The combined organic layers were dried over Na_2SO_4. After removal of ethyl acetate, the residue was purified by silica gel column chromatography (PE: EA=5:2) to remove unreacted 2′-nitrobenzaldehyde and 2′-nitroacetophenone. The crude product was dissolved in MeOH (10 mL), and $NaBH_4$ (158 mg, 4.16 mmol, 4.0 equiv.) was then added to above reaction solution at 0 °C. The mixture was stirred for 30 min at room temperature and then the solvent was removed under reduced pressure. The residue was dissolved in ethyl acetate, following by washing with saturated aqueous NH_4Cl solution and brine, respectively. The organic phase was dried over Na_2SO_4. After removal of ethyl acetate, the residue was purified by silica gel column chromatography (DCM), yielding 279 mg (17.6%). ^{1}H NMR (400 MHz, DMSO) δ 7.84 – 7.78 (m, 4H), 7.72 – 7.67 (m, 2H), 7.48 (m, 2H), 5.61 (d, J = 4.4 Hz, 2H), 5.35 – 5.01 (m, 2H), 2.12 – 1.84 (m, 2H). ^{13}C NMR (101 MHz, DMSO) δ 148.02, 147.71, 140.53, 139.34, 132.99, 132.93, 128.54, 128.32, 128.17, 127.96, 123.51, 123.42, 65.79, 64.39, 47.09, 46.86. ESI-MS: $[M-H]^-$: cacld: 317.09, found: 317.27.

*(E)-ethyl 3-(4,6-di(2-nitrophenyl)-1,3-dioxan-2-yl) acrylate (**2**).*

To the dry toluene solution (10 mL) of (E)-ethyl 4-oxobut-2-enoate (76 μL, 0.6 mmol, 1.2 equiv.) and **di-NPE** (160 mg, 0.5 mmol, 1.0 equiv.), a catalytic amount of *p*-toluenesulfonic acid (16 mg, 0.1 mmol, 0.2 equiv.) was then added. And the mixture was stirred with molecular sieves at 45 °C overnight. The solvent was removed via rotary evaporation under reduced pressure. Then the residue was diluted with ethyl acetate and washed with sodium bicarbonate. The organic layer was then dried over anhydrous Na_2SO_4. After removal of solvents, further purification by silica gel column chromatography (PE:EA = 2:1) yielded 182 mg (84.4%) of **2**. ^{1}H NMR (400 MHz, $CDCl_3$) δ 8.08 – 7.90 (m, 3H), 7.88 – 7.68 (m, 3H), 7.58 – 7.47 (m, 2H), 6.96 – 6.71 (m, 1H), 6.32 – 6.12 (m, 1H), 5.70 – 5.61 (m, 2H), 5.24 (dd, J = 3.2, 1.3 Hz), 4.28 – 4.20 (m, 2H), 2.88 – 2.74 (m, 1H), 2.48 – 2.38 (m, 1H), 1.35 – 1.28 (m, 3H). ^{13}C NMR (101 MHz, $CDCl_3$) δ 165.77, 147.37, 146.97, 141.09, 136.98, 135.85, 134.46, 133.75, 132.27, 132.17, 129.32, 129.08, 128.80, 128.71, 128.67, 128.21,

124.85, 124.56, 124.46, 124.30, 98.42, 93.42, 74.72, 70.76, 69.54, 60.81, 60.73, 39.52, 33.40, 14.19. ESI-MS: $[M+Na]^+$: cacld: 451.12, found: 451.32.

*(E)-4,4-di((2-nitrobenzyl) oxy) but-2-enoic acid (**3**)*

To a THF solution (5 mL) of **1** (347 mg, 0.84 mmol, 1.0 equiv.), NaOH (4.0 mmol, 5mL, 4.8 equiv., 2 M in H_2O) was added at 0 ºC. The mixture was allowed to slowly warm to r.t. and continued stirring overnight. After TLC analysis revealed complete conversion, the mixture was concentrated under reduced pressure. The residue was acidified with 1M NaH_2PO_4 to pH 6 and then quickly extracted with EtOAc. The combined organic layers were dried over anhydrous Na_2SO_4. After concentration under reduced pressure, the title compound was obtained as a white solid (yield: 279 mg, 95.6%). ^{1}H NMR (400 MHz, DMSO) δ 8.04 (d, *J* = 8.0 Hz, 2H), 7.74 (s, 4H), 7.57 (s, 2H), 6.64 (d, *J* = 15.8 Hz, 1H), 6.12 (d, *J* = 15.8 Hz, 1H), 5.49 (s, 1H), 4.92 (q, *J* = 14.2 Hz, 4H). ^{13}C NMR (101 MHz, DMSO) δ 167.14, 147.82, 140.91, 134.31, 133.77, 129.60, 129.24, 126.86, 124.97, 100.11, 64.82. ESI-MS: $[M-H]^-$: cacld: 387.09, found: 387.23.

*(E)-3-(4,6-bis(2-nitrophenyl)-1,3-dioxan-2-yl)acrylic acid (**4**)*

To a THF solution (5 mL) of **2** (182 mg, 0.42 mmol, 1.0 equiv.), NaOH (4.0 mmol, 5 mL, 10.0 equiv., 2 M in H_2O) was added at 0 ºC. The mixture was allowed to slowly warm to r.t. andstirred overnight. After TLC analysis revealed complete conversion, the mixture was concentrated under reduced pressure. the residue was acidified with 1 M NaH_2PO_4 to pH 6 and then quickly extracted with EtOAc. The combined organic layers were dried over anhydrous Na_2SO_4. After concentration under reduced pressure, the title compound was obtained as a white solid (yield: 155 mg, 92.4%). ^{1}H NMR (400 MHz, DMSO) δ 8.06 – 7.77 (m, 6H), 7.68 – 7.59 (m, 2H), 6.67 – 6.42 (m, 1H), 6.12 – 5.90 (m, 1H), 5.85 – 5.72 (m, 1H), 5.59 – 5.15 (m, 2H), 2.86 – 2.53 (m, 1H), 2.41 – 2.33 (m, 1H). ^{13}C NMR (101 MHz, DMSO) δ 167.00, 166.90, 150.33, 147.97, 147.69, 141.29, 136.12, 135.18, 134.70, 134.29, 132.62, 131.71, 130.19, 129.70,

129.53, 128.78, 128.60, 125.33, 125.05, 124.83, 124.68. 98.06, 93.37, 74.07, 70.64, 69.86, 32.41. ESI-MS: $[M-H]^-$: cacld: 399.09, found: 399.19.

*(E)-2, 5-dioxopyrrolidin-1-yl 4, 4-di((2-nitrobenzyl)oxy)but-2-enoate (**5**)*

Dicyclohexylcarbodiimide (DCC) (149 mg, 0.72 mmol, 1.0 equiv.) in 5mL THF was added dropwise to a solution of **3** (279 mg, 0.72 mmol, 1.0 equiv.) and *N*-hydroxysuccinimide (NHS) (83 mg, 0.72 mmol, 1.0 equiv.) in 5 mL THF at room temperature. The mixture was stirred overnight. After filtration, the filtrate was concentrated to give yellow solid. The crude product was washed with sodium chloride solution and extracted with EtOAc. After removal of solvents, further purification by silica gel column chromatography (DCM: EA=20:1) yielded 210 mg (60.1%) of **5** as a light yellow solid. ^{1}H NMR (400 MHz, $CDCl_3$) δ 8.10 (d, *J* = 8.0 Hz, 2H), 7.80 (d, *J* = 7.8 Hz, 2H), 7.68 (t, *J* = 7.6 Hz, 2H), 7.49 (t, *J* = 7.6 Hz, 2H), 7.22 (dd, *J* = 16.0, 3.6 Hz, 1H), 6.54 (d, *J* = 16.0 Hz, 1H), 5.56 (d, *J* = 3.6 Hz, 1H), 5.07 (q, *J* = 14.8 Hz, 4H), 2.89 (s, 4H). ^{13}C NMR (101 MHz, $CDCl_3$) δ 168.92, 160.68, 147.41, 147.20, 133.89, 133.55, 128.81, 128.48, 124.89, 119.54, 98.89, 64.95, 25.63. ESI-MS: $[M+Na]^+$: cacld: 508.11, found: 508.32.

*(E)-2,5-dioxopyrrolidin-1-yl 3-(4,6-bis(2-nitrophenyl)-1,3-dioxan-2-yl)acrylate(**6**).*

DCC (58 mg, 0.28 mmol, 1.0 equiv.) in 5 mL THF was added dropwise to a solution of **4** (112 mg, 0.28 mmol, 1.0 equiv.) and NHS (32 mg, 0.28 mmol, 1.0 equiv.) in 5mL THF at room temperature. The mixture was stirred overnight. After filtration, the filtrate was concentrated to give yellow solid. The crude product was washed with sodium chloride solution and extracted with EtOAc. After removal of solvents, further purification by silica gel column chromatography (PE: EA=1:2) yielded 120 mg (86.2%) of **6** as a light yellow solid. ^{1}H NMR (400 MHz, $CDCl_3$) δ 8.05-7.68 (m, 6H), 7.53 – 7.47 (m, 2H), 7.18 – 6.92 (m, 1H), 6.50 – 6.29 (m, 1H), 5.97 – 5.26 (m, 3H), 2.86 – 2.84 (m, 4H), 2.76 – 2.38 (m, 1H) 2.03 – 1.71 (m, 1H).^{13}C NMR (101 MHz, $CDCl_3$) δ 160.85, 150.03, 147.27, 146.91, 146.76, 146.68, 136.73, 135.65, 134.56, 133.87, 132.32, 131.72, 129.50, 129.07, 128.94, 128.82, 128.64, 128.19, 124.86,

124.60, 118.50, 118.34, 97.52, 92.66, 74.86, 70.96, 69.77, 39.57, 33.28, 25.63, 25.62. ESI-MS: [M+Na]$^+$: cacld: 520.11, found: 520.34.

Synthesis of cross-linked adducts

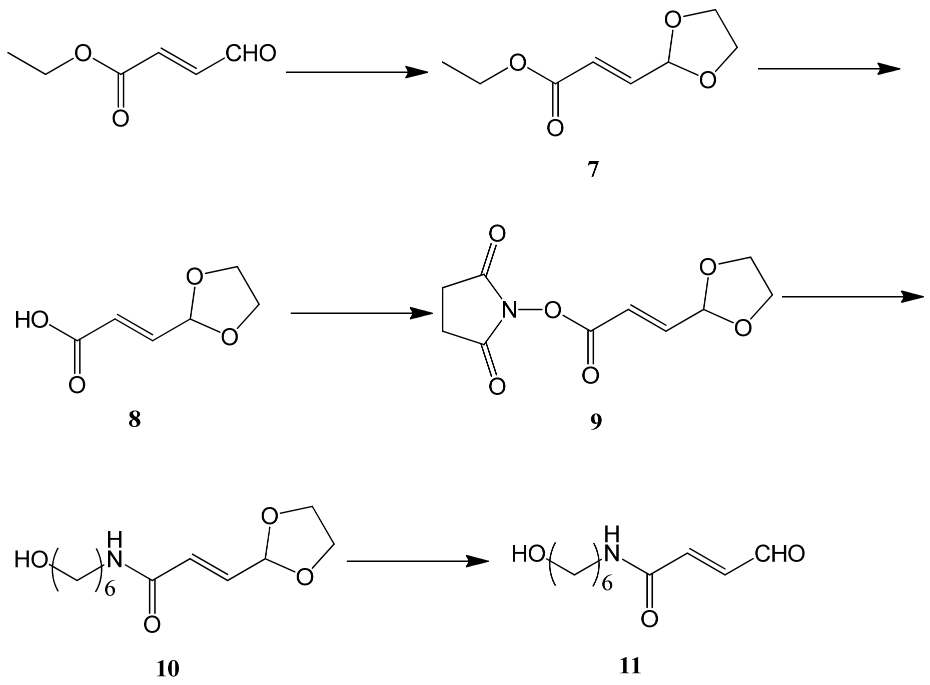

Scheme S2

(E)-ethyl 3-(1,3-dioxolan-2-yl)acrylate (7).

First, (E)-ethyl 4-oxobut-2-enoate (1.1 mL, 8.0 mmol, 1.0 equiv.) and ethylene glycol (535 μL, 9.6 mmol, 1.2 equiv.) were dissolved in 15 mL of dry toluene. A catalytic amount of *p*-toluenesulfonic acid (138 mg, 0.8 mmol, 0.1 equiv.) was then added to the mixture, and and the mixture was stirred with molecular sieves at 45 ^{o}C overnight. The solvent was removed via rotary evaporation under reduced pressure. And the residue was washed with sodium bicarbonate and extracted with ethyl acetate. Subsequent purification by column chromatography (PE:EA=10:1) yielded 757 mg (55.5%) of **7** as a yellow oil. ^{1}H NMR (400 MHz, $CDCl_3$) δ 6.76 (dd, *J* = 15.8, 4.7 Hz, 1H), 6.13 (d, *J* = 15.8 Hz, 1H), 5.44 (d, *J* = 4.7 Hz, 1H), 4.20 (q, *J* = 7.1 Hz, 2H), 4.08 – 3.87 (m, 4H), 1.28 (t, *J* = 7.1 Hz, 3H). ^{13}C NMR (101 MHz, $CDCl_3$) δ 165.74, 141.88, 124.46, 101.26, 65.10, 60.70, 14.17. ESI-MS: [M+Na]$^+$: cacld: 195.07, found: 195.09.

*(E)-3-(1,3-dioxolan-2-yl)acrylic acid (**8**).*

First, **7** (757 mg, 4.4 mmol, 1.0 equiv.) was dissolved in 5mL THF and NaOH (12.0 mmol, 15 mL, 3.4 equiv., 2 M in H_2O) was then added at 0 ºC. The mixture was allowed to slowly warm to r.t. and was stirred overnight. After TLC analysis revealed complete conversion, the mixture was concentrated under reduced pressure and the residue was acidified with 1 M NaH_2PO_4 until pH 6. After quick extraction with EtOAc, the organic layers were combined, dried over Na_2SO_4 and concentrated under reduced pressure, yielding the title compound as a white solid (yield: 524 mg, 82.6%). ^{1}H NMR (400 MHz, DMSO) δ 12.62 (s, 1H), 6.57 (dd, *J* = 15.7, 5.4 Hz, 1H), 6.07 (dd, *J* = 15.7, 0.8 Hz, 1H), 5.41 (dd, *J* = 5.4, 0.6 Hz, 1H), 3.95 – 3.84 (m, 4H). ^{13}C NMR (101 MHz, DMSO) δ 166.97, 142.19, 125.60, 100.96, 65.00. ESI-MS: $[M-H]^-$: cacld: 143.04, found: 142.94.

*(E)-2, 5-dioxopyrrolidin-1-yl 3-(1,3-dioxolan-2-yl) acrylate (**9**).*

DSC (1.86 g, 7.3 mmol, 2.0 equiv.) in 5 mL CH_3CN was added dropwise to a solution of **8** (524 mg, 3.6 mmol, 1.0 equiv.), DMAP (44 mg, 0.36 mmol, 0.1 equiv.) and TEA (1.0 mL, 7.3 mmol, 2.0 equiv.) in 5 mL CH_3CN at room temperature. The mixture was stirred overnight. The mixture was then concentrated to give yellow solid. The crude product was washed with sodium chloride solution and extracted with EtOAc. Subsequent purification by column chromatography (EA: MeOH=10:1) yielded 757 mg (86.3%) of **9** as a light yellow solid. ^{1}H NMR (400 MHz, $CDCl_3$) δ 7.06 (dd, *J* = 15.8, 3.7 Hz, 1H), 6.34 (dd, *J* = 15.8, 1.1 Hz, 1H), 5.54 (dd, *J* = 3.6, 0.9 Hz, 1H), 4.00 – 3.97 (m, 4H), 2.85 (s, 4H). ^{13}C NMR (101 MHz, $CDCl_3$) δ 169.08, 168.92 (s), 147.93 (s), 117.97 (s), 100.48 (s), 65.21 (s), 25.61 (s). ESI-MS: $[M+H]^+$: cacld: 241.06, found: 241.31.

*(E)-3-(1,3-dioxolan-2-yl)-N-(6-hydroxyhexyl)acrylamide (**10**)*

6-aminohexan-1-ol (44 mg, 0.38 mmol, 1.0 equiv.) was added to the solution of **9** (108 mg, 0.45 mmol, 1.2 equiv.) in 5 mL CH_3CN at room temperature. The mixture

was stirred for 2 h. After the completion of reaction, the reaction mixture was concentrated under reduced pressure and the residue was extracted with EtOAc and washed with sodium chloride solution. Subsequent purification by column chromatography (EA: MeOH = 10:1) yielded 90 mg (98.8%) of **10** as a light yellow solid. ^{1}H NMR (400 MHz, $CDCl_3$) δ 6.66 (dd, *J* = 15.4, 4.6 Hz, 1H), 6.11 (d, *J* = 15.5 Hz, 1H), 5.63 (s, 1H), 5.44 (d, *J* = 4.6 Hz, 1H), 4.05 – 3.87 (m, 4H), 3.64 (t, *J* = 6.4 Hz, 2H), 3.33 (dd, *J* = 13.2, 6.8 Hz, 2H), 1.56 (m, *J* = 14.4, 7.1 Hz, 7H), 1.46 – 1.31 (m, 5H).^{13}C NMR (101 MHz, $CDCl_3$) δ 164.82 (s), 137.82 (s), 126.83 (s), 101.55 (s), 65.04 (s), 62.69 (s), 39.49 (s), 32.52 (s), 29.53 (s), 26.48 (s), 25.26 (s). ESI-MS: $[M+Na]^+$: cacld: 266.15, found: 266.22.

*(E)-N-(6-hydroxyhexyl)-4-oxobut-2-enamide (**11**).*

10 (90 mg, 0.37 mmol, 1.0 equiv.) was suspended in 8 mL of a 3:1 mixture of acetone and water. Then a catalytic amount of para-TsOH (5 mg) was added and the suspension was stirred overnight at ambient temperature. The mixture was concentrated under reduced pressure and the residue was extracted with ethyl acetate. The combined organic layers were dried over Na_2SO_4. After solvents were evaporated, further purification with silica gel chromatography (DCM: EA = 1:1) afforded the desired product as a yellow solid (46 mg, 62.5%). ^{1}H NMR (400 MHz, $CDCl_3$) δ 9.72 (d, *J* = 7.4 Hz, 1H), 6.92 (dd, *J* = 15.6, 7.4 Hz, 1H), 6.74 (d, *J* = 15.6 Hz, 1H), 3.64 (t, *J* = 6.4 Hz, 2H), 3.38 (q, *J* = 6.4 Hz, 2H), 1.62 – 1.54 (m, 4H), 1.40 – 1.37 (m, 4H).^{13}C NMR (101 MHz, $CDCl_3$) δ 192.15, 163.16, 141.98, 137.08, 62.33, 39.60, 32.12, 28.98. 26.19, 24.96. ESI-MS: $[M-H]^-$: cacld: 198.12, found: 198.17.

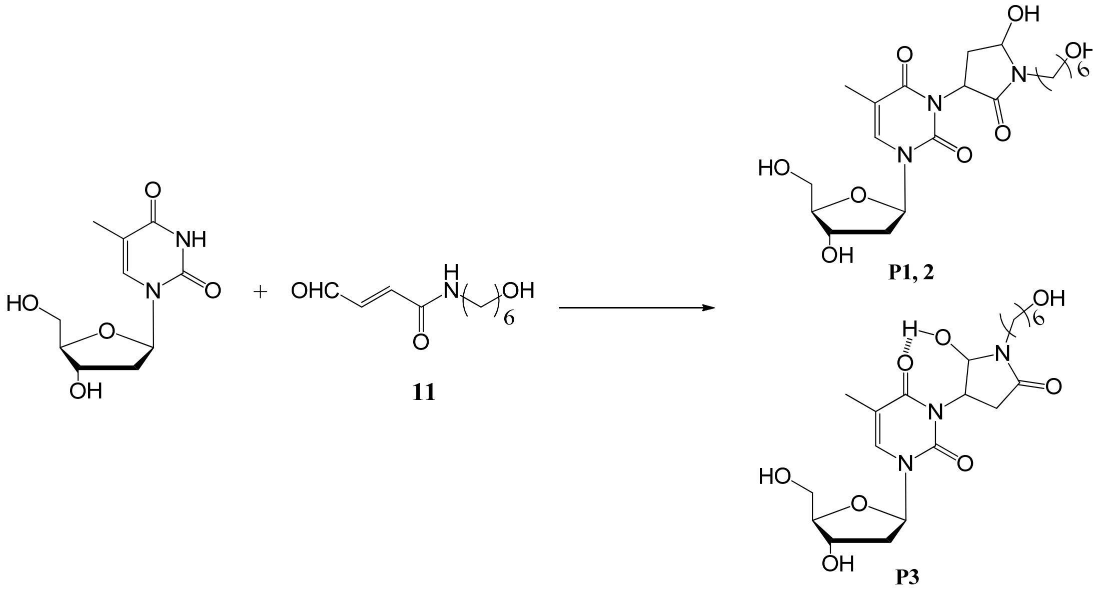

Scheme S3

Synthesis of the authentic adducts with 11

The DMF solution of **11** (46 mg, 0.23 mmol, 1.0 equiv.) was added to the solution of thymine (223 mg, 0.92 mmol, 4.0 equiv.) in 1.0 mL PBS buffer (10 mM, pH 7.0). The mixture was shaken in an Eppendorf Thermomixer Comfort at 1000 rpm and 37ºC for 4 d. The crude products were obtained after the removal of most of unreacted thymine with silica gel chromatography (DCM: MeOH = 10:1) and were purified by RP-HPLC. The running conditions: Phase A, 0.05 mM TEAA; B, MeCN. The following step-wise gradient was used with flow rate of 1 mL/min, 0-10% MeCN in 30 min. The column temperature was maintained at 50ºC.

P1, 2

^{1}H NMR (400 MHz, D_2O) δ 7.64 (s, 1H), 6.25 (d, *J* = 24.8 Hz, 1H), 5.59 – 5.38 (m, 1H), 5.33 (s, 1H), 4.42 (s, 1H), 3.99 (s, 1H), 3.83 – 3.67 (m, 2H), 3.58 – 3.55 (t, *J* = 4.7 Hz, 2H), 3.42 – 3.29 (m, 2H), 2.85 (s, 1H), 2.36 – 2.32 (m, 2H), 2.03 – 2.01 (m, 1H), 1.91 (s, 1.5H), 1.86 (s, 1.5H), 1.60 – 1.51 (m, 4H), 1.32 (s, 4H).^{13}C NMR (101 MHz, $CDCl_3$) δ 171.91, 171.77, 165.28, 164.32, 151.97, 135.92, 110.85, 110.48, 86.55, 86.17, 85.58, 80.21, 80.07, 70.25, 70.20, 62.46, 61.70, 61.02, 52.27, 51.08, 40.32, 40.26, 38.66, 31.37, 31.29, 31.13, 26.46, 26.40, 25.84, 24.64, 12.40, 11.86.

ESI-MS: [M+Na] $^+$: cacld: 464.21, found: 464.60.

P3

^{1}H NMR (400 MHz, D_2O) δ 7.68 (s, 1H), 6.32 – 6.19 (m, 1H), 5.78 (dt, *J* = 8.6, 66.4 Hz, 1H), 5.48 (d, *J* = 6.7 Hz, 1H), 4.46 (s, 1H), 4.03 (s, 1H), 3.88 – 3.72 (m, 2H), 3.61 (d, *J* = 5.0 Hz, 2H), 3.49 (d, *J* = 5.4 Hz, 1H), 3.23 (d, *J* = 9.0 Hz, 1H), 2.60 (s, 1H), 2.38-2.35 (m, 2H), 2.35– 2.29 (m, 1H), 1.95 (s, 1.5H), 1.88 (s, 1.5H), 1.66 – 1.58 (m, 4H), 1.39 (s, 4H).^{13}C NMR (101 MHz, $CDCl_3$) δ 173.13, 172.89, 165.34, 164.44, 151.93, 150.25, 136.15, 136.03, 110.82, 110.46, 86.66, 86.48, 85.81, 81.07, 70.34, 61.79, 61.19, 61.13, 52.41, 51.20, 40.67, 40.56, 38.74, 31.39, 31.24, 31.21, 26.29, 26.17, 25.84, 24.70, 12.43, 11.88. ESI-MS: [M+Na] $^+$: cacld: 464.21, found: 464.56.

Figure S1. RP-HPLC purification of ODN**1**.

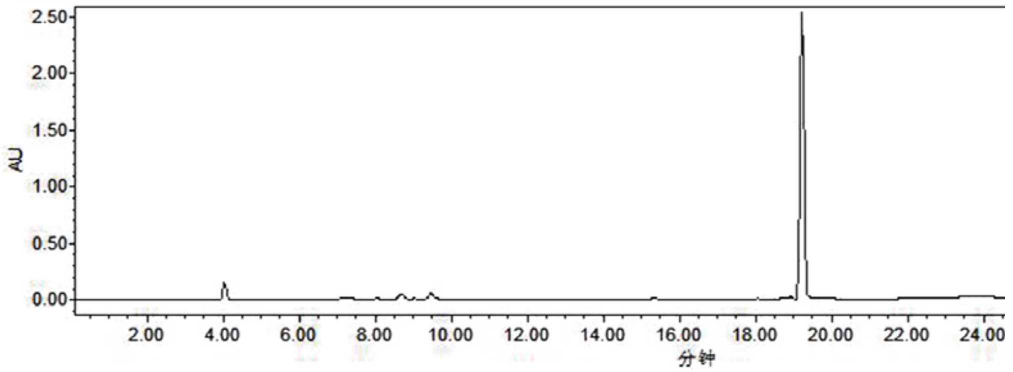

Figure S2. RP-HPLC analysis of deprotection of ODN**1** under different UV irradiation time.

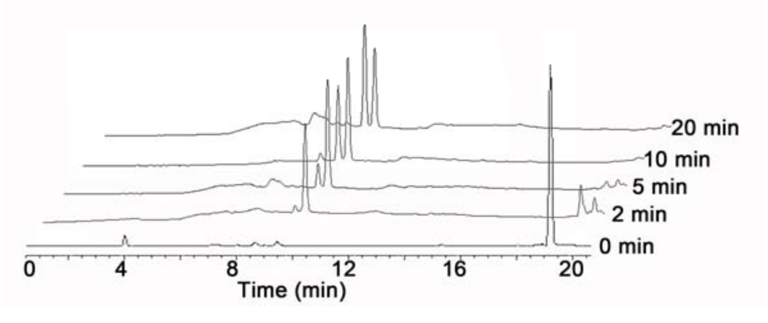

Figure S3. Gel-shifts and RP-HPLC analysis of ODN**1** self-cross-linking reaction. (a) ODN1 after UV irradiation for 5 min, the deprotected ODN1 after incubation at 37ºC, pH 7.0 for 0 h, 2 h, 12 h, 24 h. (b) (i) ODN1 after UV irradiation for 5 min ; (ii) The deprotected ODN1 after incubation at 37ºC, pH 7.0 for 24 h

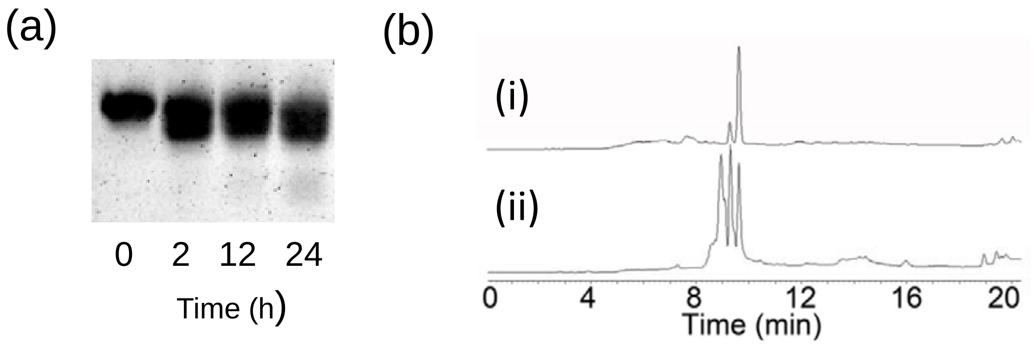

Figure S4. ESI-MS of the self cross-links.

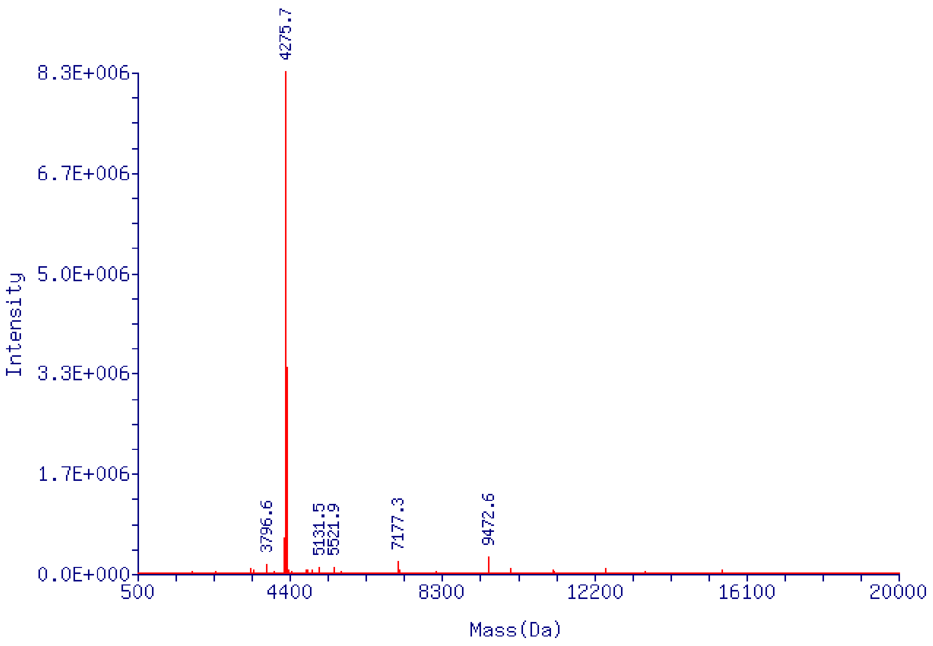

Figure S5. Gel-shift analysis of cross-linking reaction with ODN**1** and ODN**4** without UV irradition. Cross-linking conditions: [ODN**1**] = 10 μM, ODN**4** = 1 μM, 10 mM PBS buffer, 100 mM NaCl, 37 ºC, pH 7.0. The reaction was followed by electrophoresis using 15% denatured polyacrylamide gel. No cross-linked products were observable. CON=control

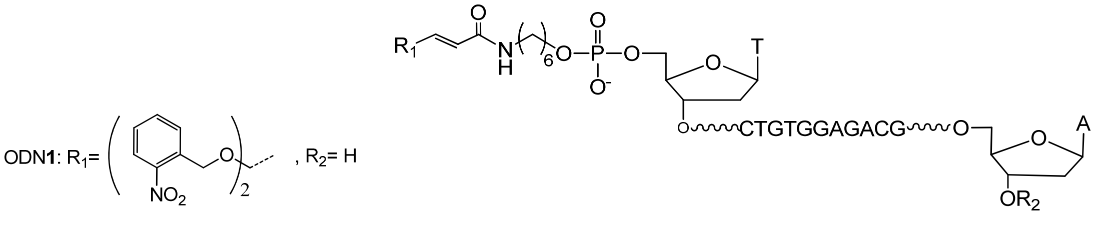

ODN**4**-FAM: 3'-XXXXAGACACCTCTGCT (FAM) -5'

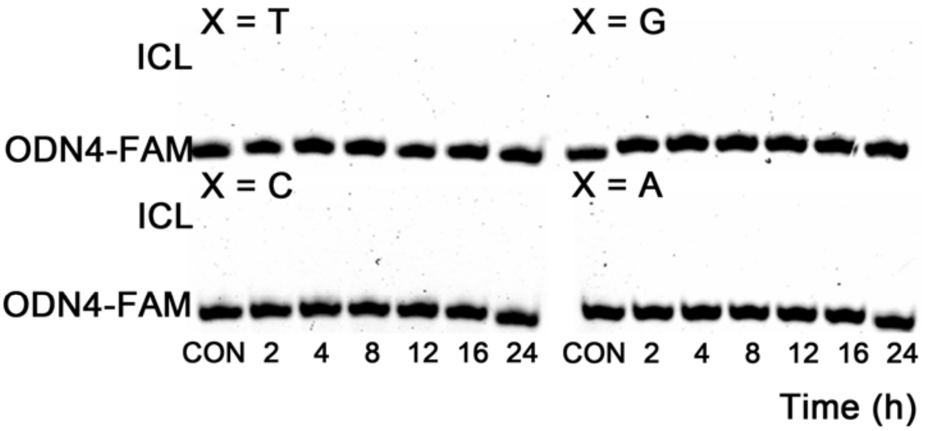

Figure S6. Cross-linking reaction with ODN**2** and ODN**4** (X = T, G). Cross-linking conditions: ODN**2**= 10 µM, ODN**4** = 1 µM, 10 mM MES buffer, 100 mM NaCl, 37 ºC, pH 5.0; 10 mM carbonate buffer, pH 9.0. The reaction was followed by electrophoresis using 15% denatured polyacrylamide gel. The fast- and slow-moving bands represent ONN**2** and the cross-linked products, respectively. The cross-link yield was obtained by quantification of the bands by FAM fluorescence, and it was plotted against time. (a) Yields obtained with ODN**4** (X = T). (b) Gel-shift analysis of the reaction with ODN**4** (X = T). (c) Yields obtained with ODN**4** (X = G). (d) Gel-shift analysis of the reaction with ODN**4** (X = G). CON=control

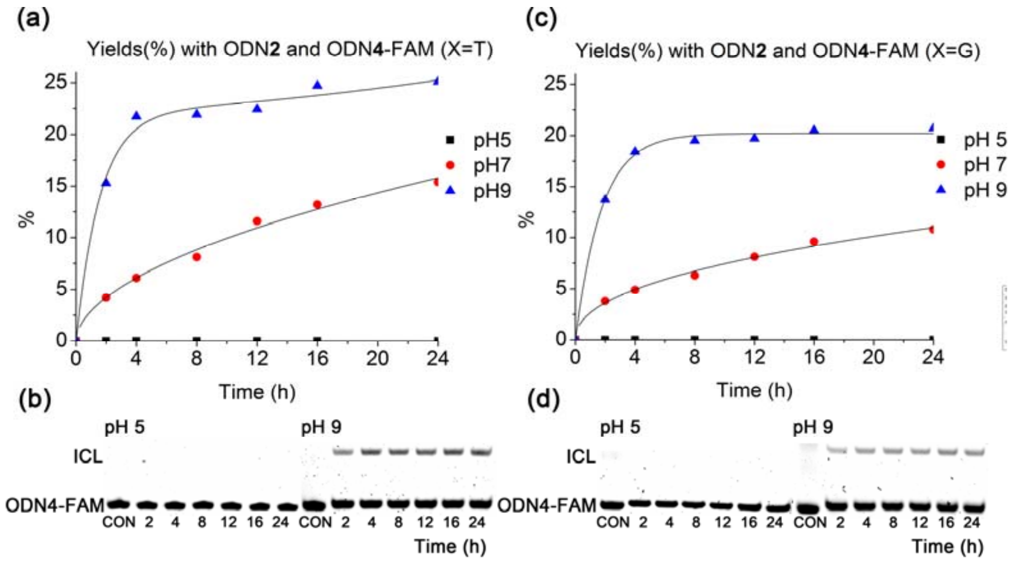

Figure S7. Cross-linking reaction with ODN**1** and ODN**4**. Cross-linking conditions: ODN**1** = 10 μM, ODN**4** = 1 μM, 10 mM PBS buffer, 100 mM NaCl, 37 ºC or 60 ºC, pH 7.0. The reaction was followed by electrophoresis using 15% denatured polyacrylamide gel. The fast- and slow-moving bands represent RNA1and the cross-linked products, respectively. The cross-link yield was obtained by quantification of the bands by FAM fluorescence, and it was plotted against time. (a) Yields obtained with ODN**1** and ODN**4** (X = T). (b) Gel-shift analysis of the reaction with ODN**1**and ODN**4** (X = T). (c) Yields obtained with ODN**1** and ODN**4** (X = G). (d) Gel-shift analysis of the reaction with ODN**1** and ODN**4** (X = G). CON=control

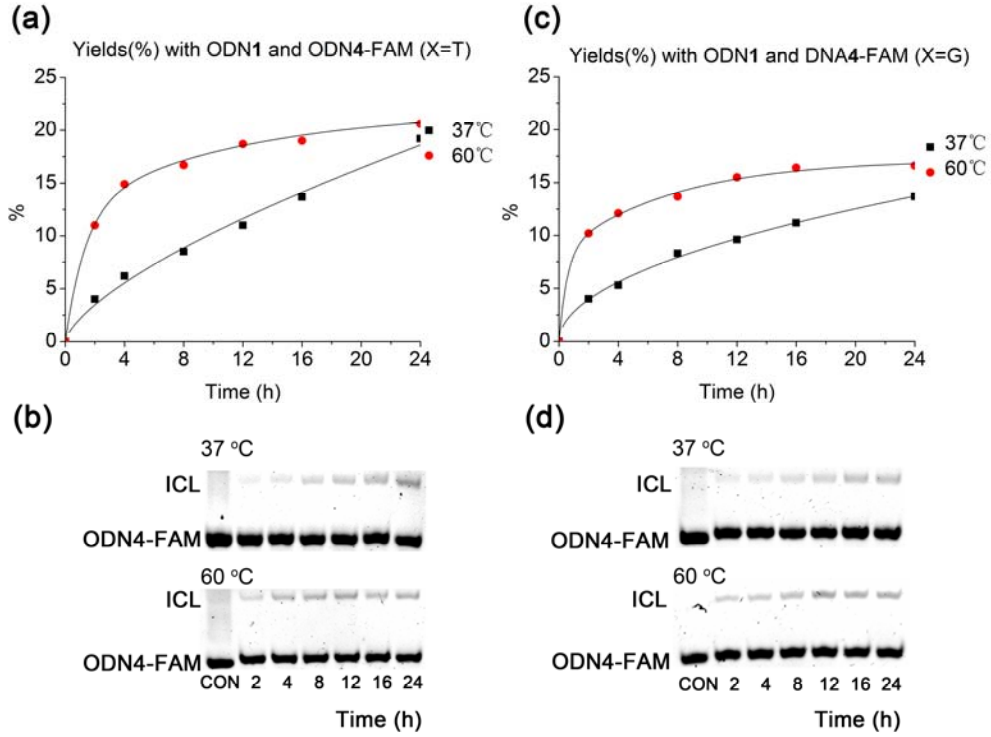

Figure S8. Cross-linking reaction with ODN**2** and ODN**4**. Cross-linking conditions: ODN**2** = 10μM, ODN**4** = 1μM, 10 mM PBS buffer, 100 mM NaCl, 37 ºC or 60 ºC, pH 7.0. The reaction was followed by electrophoresis using 15% denatured polyacrylamide gel. The fast- and slow-moving bands represent ODN**2** and the cross-linked products, respectively. The cross-link yield was obtained by quantification of the bands by FAM fluorescence, and it was plotted against time. (a) Yields obtained with ODN**2** and ODN**4** (X = T). (b) Gel-shift analysis of the reaction with ODN**2** and ODN**4** (X = T). (c) Yields obtained with ODN**2** and ODN**4** (X = G). (d) Gel-shift analysis of the reaction with ODN**2** and ODN**4** (X = G). CON=control

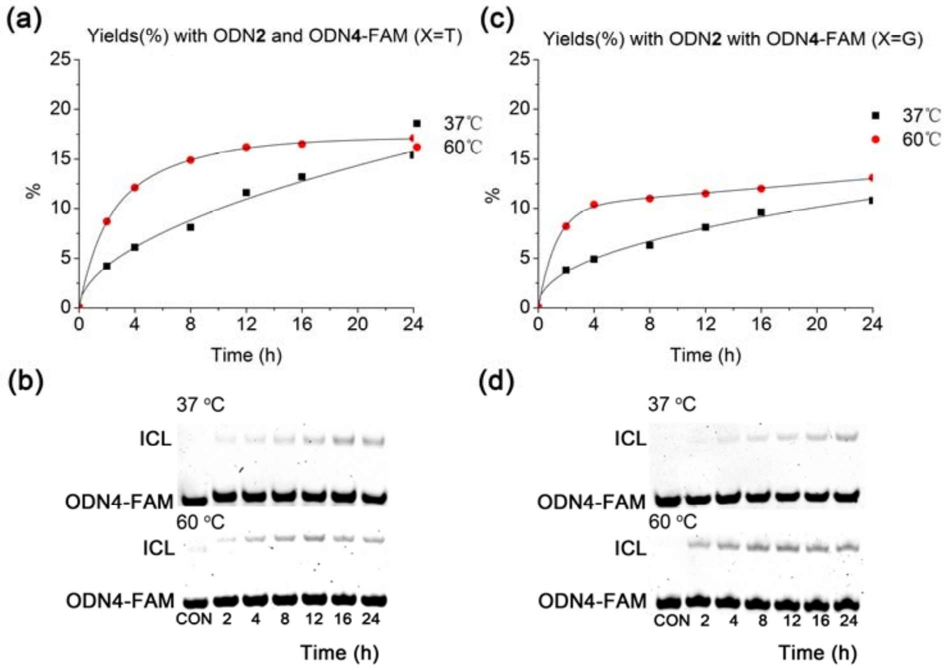

Figure S9. Cross-linking reaction with ODN**3** and ODN**5, 6, 7, 8**. Cross-linking conditions: ODN**3** = 10 μM, [ODN**5, 6, 7, 8**] = 1 μM, 10 mM PBS buffer, 100 mM NaCl, 37 ºC, pH 7.0. The reaction was followed by electrophoresis using 15% denatured polyacrylamide gel. The fast- and slow-moving bands represent ODN**5**, **6**, **7**, **8** and the cross-linked products, respectively. The cross-link yield was obtained by quantification of the bands by FAM fluorescence, and it was plotted against time. (a) Yields obtained with ODN**3**. (b) Gel-shift analysis of the reaction with ODN**3**. CON=control

ODN**5**: 3'-CCCTAGACACCTCTGCTTTTTT (FAM)-5'
ODN**6**: 3'-CCTCAGACACCTCTGCTTTTTT (FAM)-5'
ODN**7**: 3'-CTCCAGACACCTCTGCTTTTTT (FAM)-5'
ODN**8**: 3'-TCCCAGACACCTCTGCTTTTTT (FAM)-5'

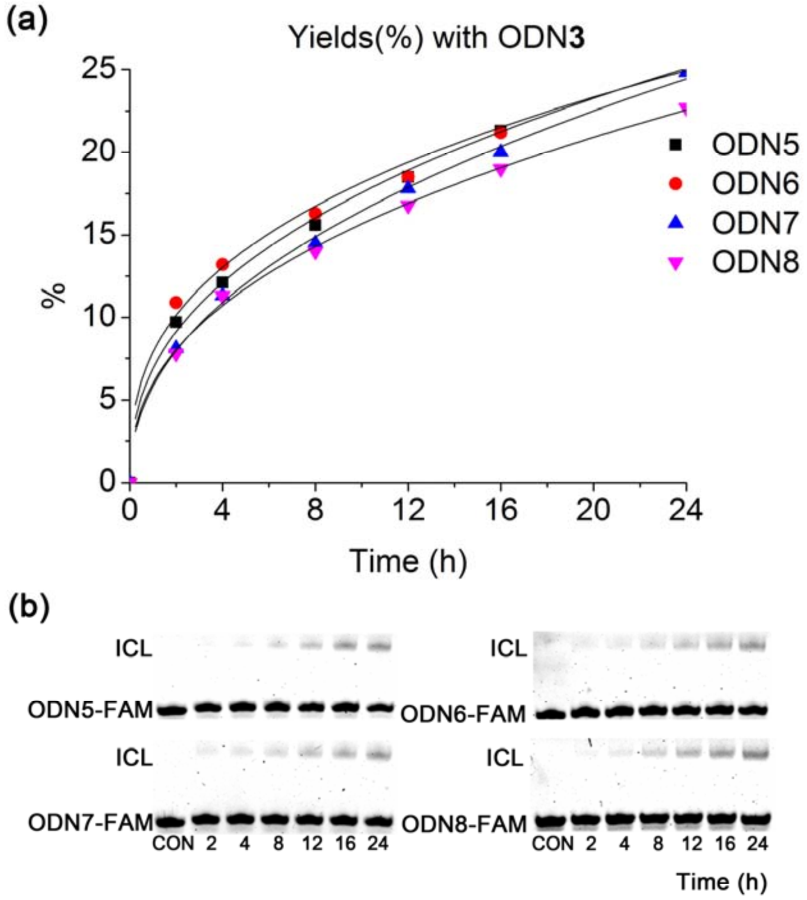

Figure S10. RP-HPLC analysis of cross-linking reaction with ODN**1** and ODN**4** (X = T). (a) Cross-linking conditions: [ODN**1**] = 1 μM, ODN**4** = 1 μM, 10 mM PBS buffer, 100 mM NaCl, 37 ºC, pH 7.0, after incubation for 2h. (de-ODN**1** = deprotected ODN**1**). (b) After incubation for 24 h. (c) ICL product purified by Gel. The running conditions: Phase A, 0.05 mM TEAB; Phase B, MeCN. The following step-wise gradient was used with flow rate of 1 mL/min, 0-20% MeCN in 15 min. The column temperature was maintained at 40 ºC.

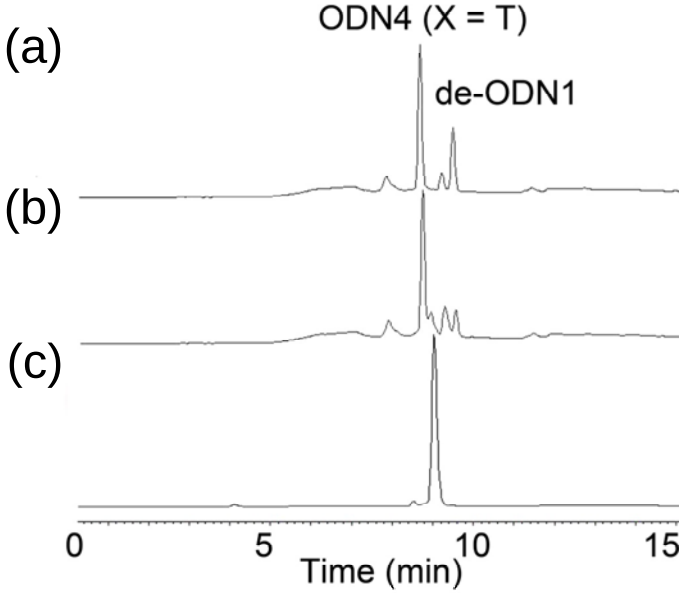

Figure S11. ESI-MS of the cross-linked duplex (X=T).

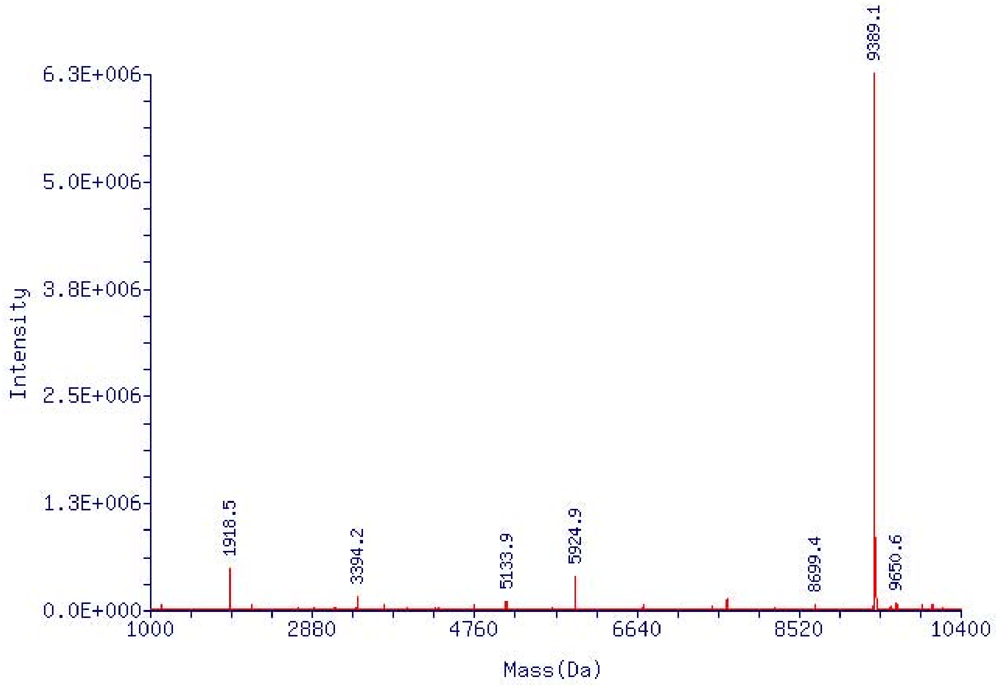

Figure S12. ESI-MS of the cross-linked duplex (X=G).

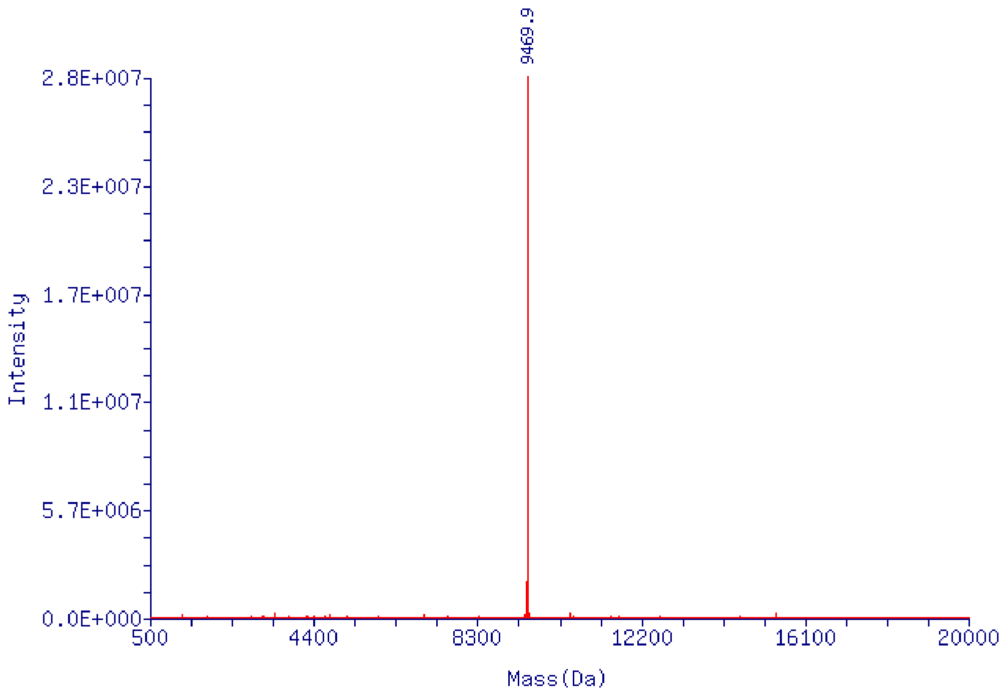

Figure S13. (a) HPLC analysis of the enzymatic hydrolyzates of unmodified oligodexynucleotides ODN4 (X = T) for negative control experiment. (b) HPLC analysis of the enzymatic hydrolyzates of the cross-linked products (X=T) obtained at pH 9.0. (c) HPLC analysis of the enzymatic hydrolyzates of the cross-linked products (X=T) obtained at pH 7.0

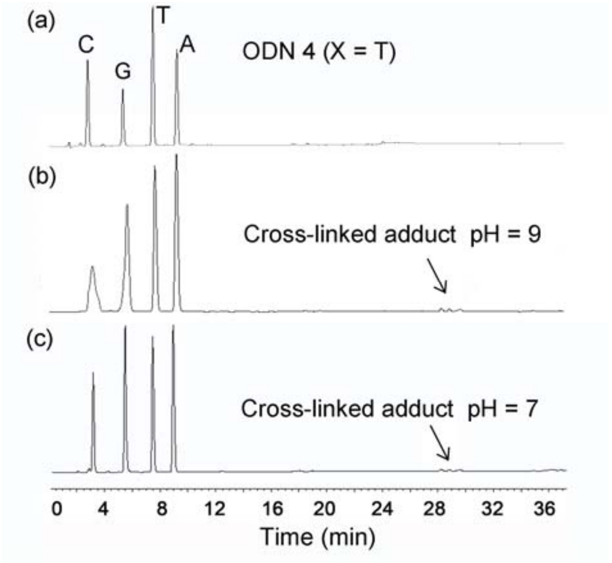

Figure S14. (a) HPLC analysis of the enzymatic hydrolyzates of unmodified oligodexynucleotides ODN4 (X = T) for negative control experiment. (b) HPLC analysis of the enzymatic hydrolyzates of the cross-linked products (X= G) obtained at pH 9.0.

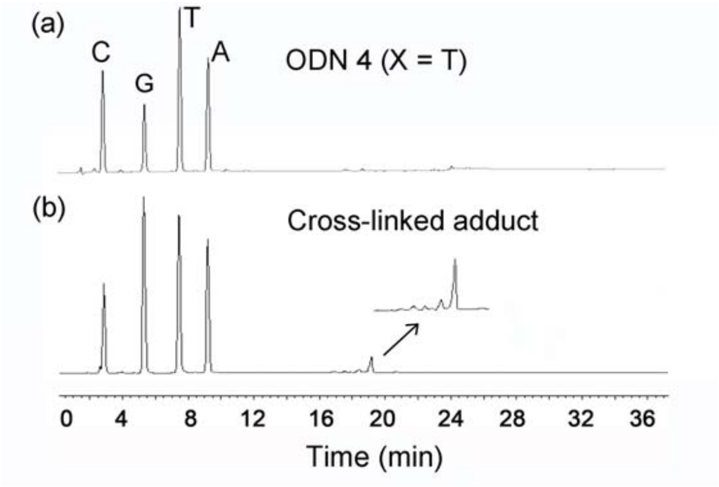

Figure S15. Cross-linking reaction with ODN1 and ODN4 (X = T). (a) Cross-linking conditions: ODN1 = 10 µM, ODN4 = 1 µM, 10 mM PBS buffer, 100 mM NaCl, 37°C, pH 7.0. (b) Cross-linking conditions: ODN1 = 10 µM, ODN4 = 1 µM, 10 mM PBS buffer, 100 mM NaCl, 0.05% 2,2,6,6-Tetramethyl-1-piperidinyloxy (TEMPO), 37°C, pH 7.0. The reaction was followed by electrophoresis using 20 % denatured polyacrylamide gel. The fast- and slow-moving bands represent ODN4 and the cross-linked products, respectively.

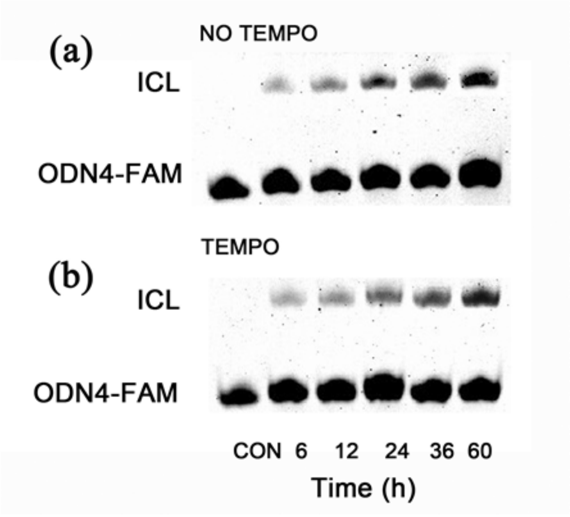

Figure S16. Cross-linking reaction with ODN**1** and ODN**4** (X = C, A). Cross-linking conditions: ODN**1** = 10 μM, ODN**4** = 1 μM, 10 mM MES buffer, 100 mM NaCl, 37 ºC, pH 5.0; 10 mM carbonate buffer, pH 9.0. The reaction was followed by electrophoresis using 15% denatured polyacrylamide gel. The fast- and slow-moving bands represent ODN**4** and the cross-linked products, respectively. The cross-link yield was obtained by quantification of the bands by FAM fluorescence, and it was plotted against time. (a) Gel-shift analysis of the reaction with ODN**4** (X = C, A) at pH 5.0. (b) Gel-shift analysis of the reaction with ODN**4** (X = C, A) at pH 9.0.

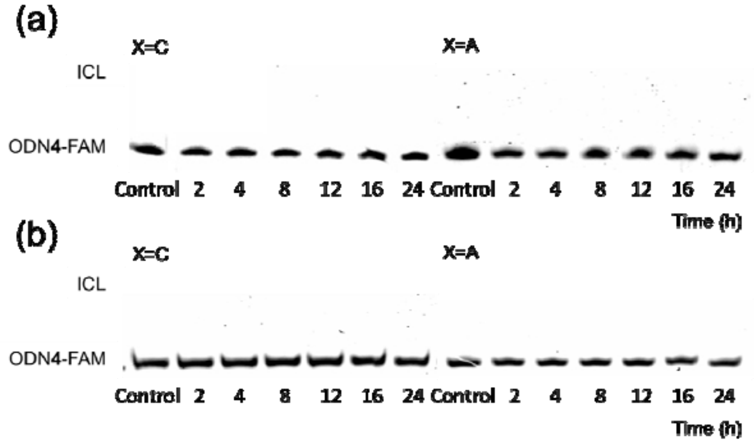

Figure S17. ^{1}H-^{1}H-COSY of the authentic adducts (**P3**).

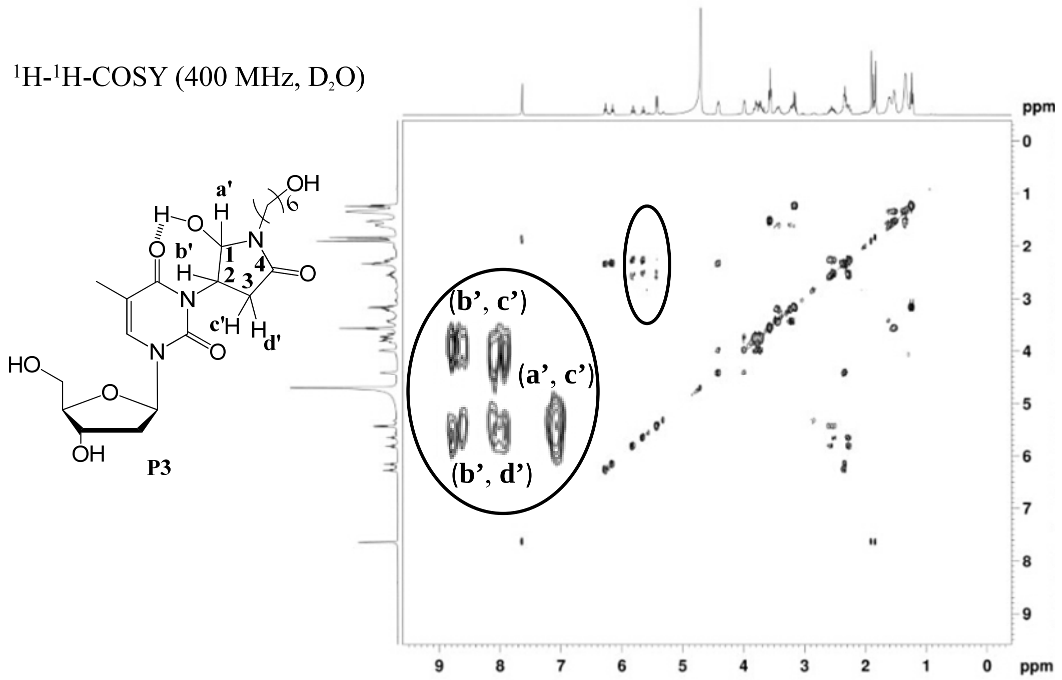

Figure S18. NMR and MS of synthetic compounds and oligonucleotides

NMR of Compound **di-NPE**

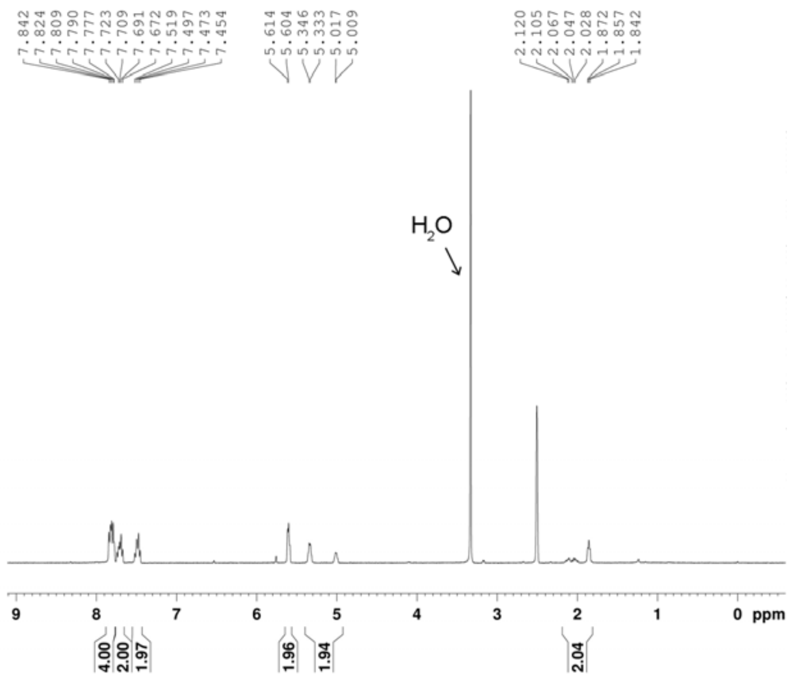

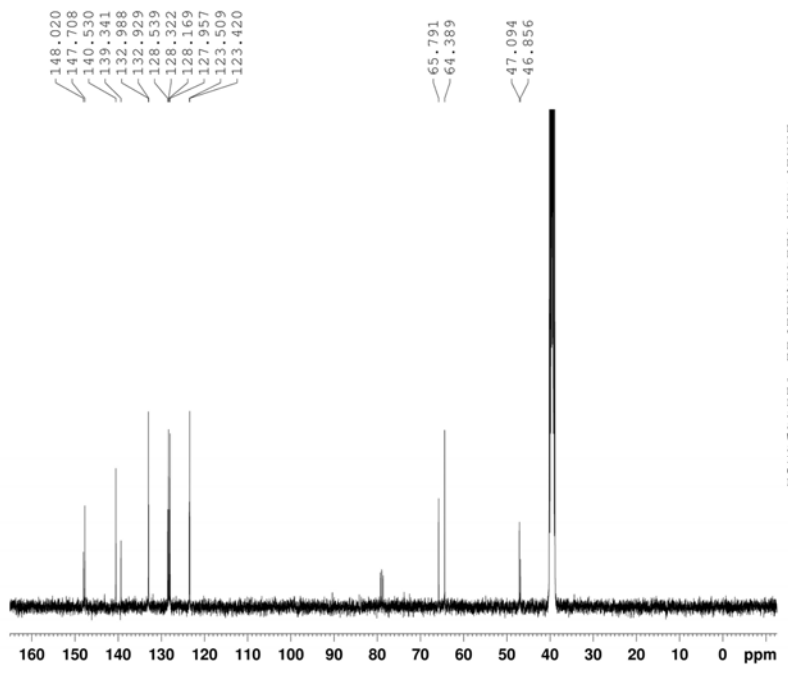

NMR of Compound **1**

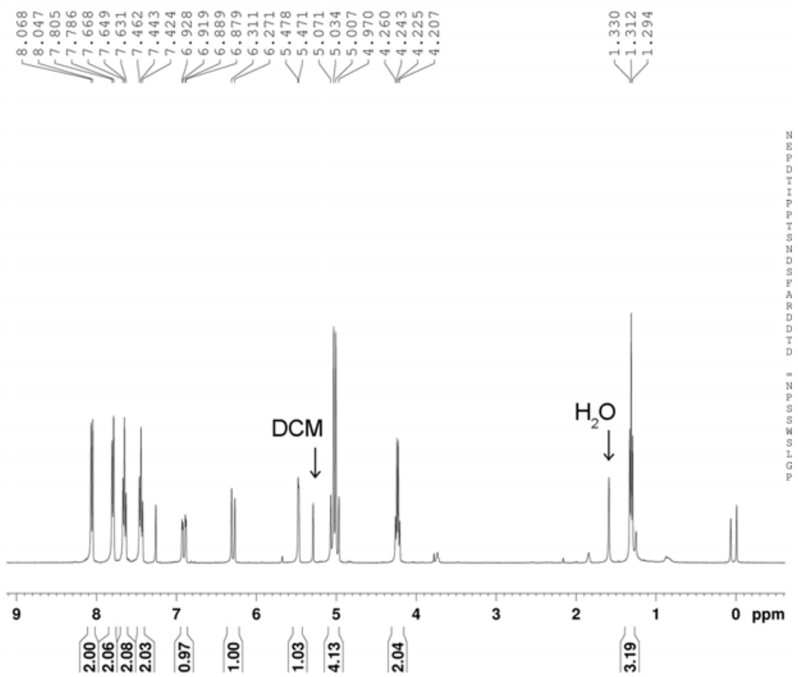

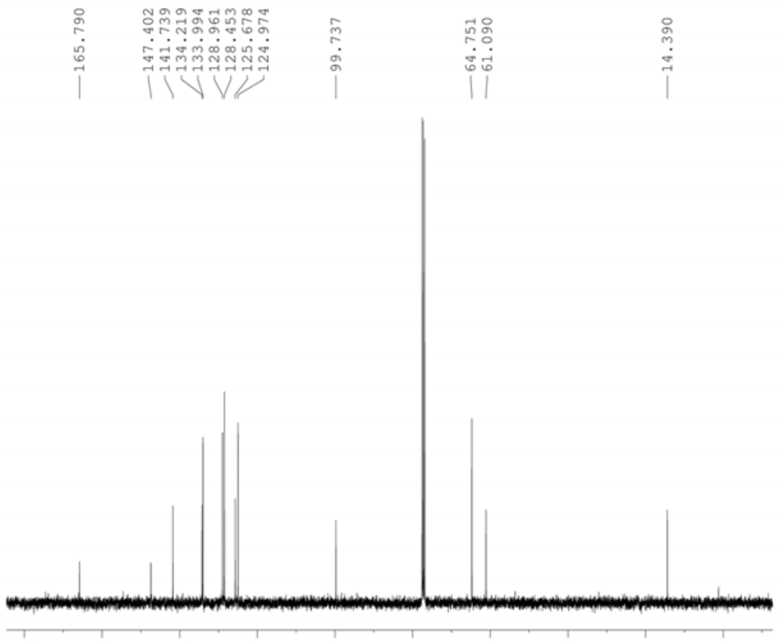

NMR of Compound **2**

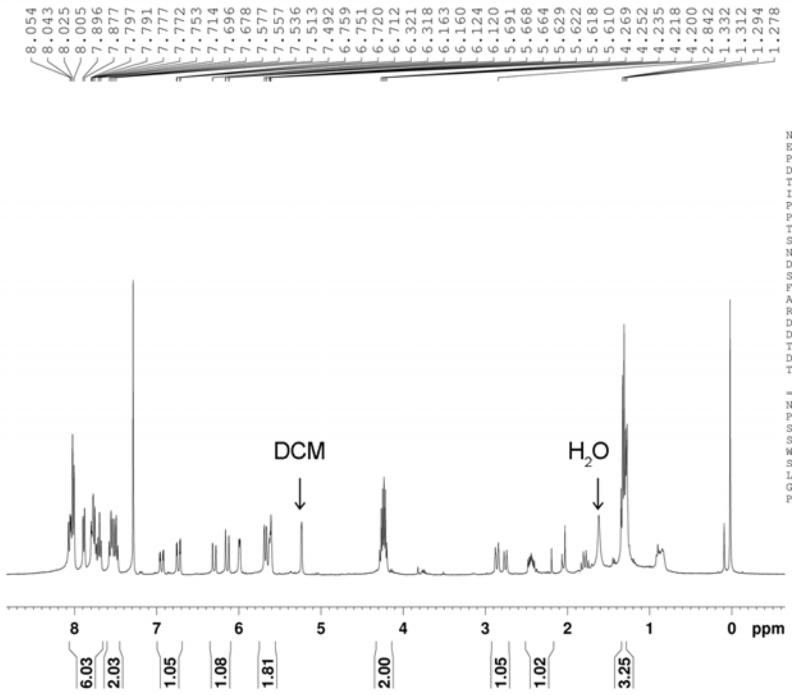

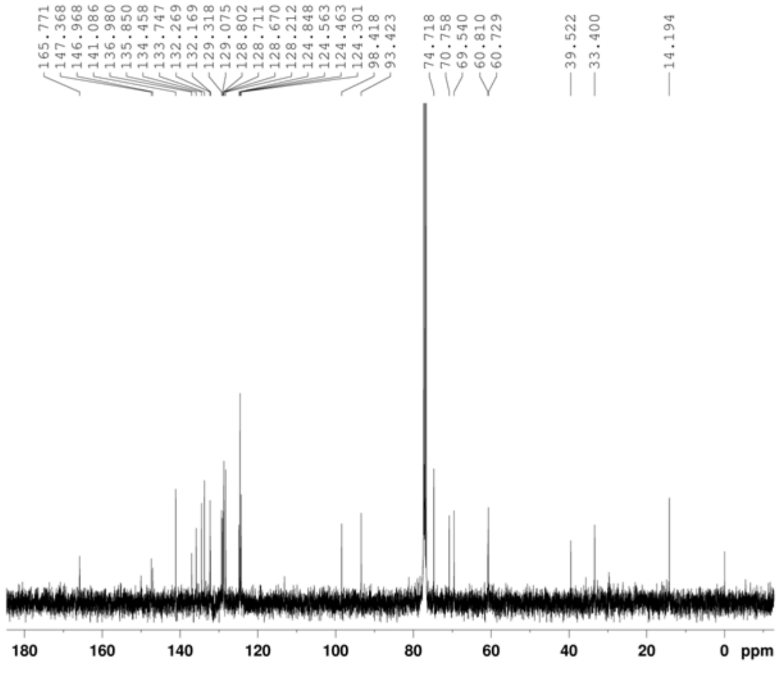

NMR of Compound **3**

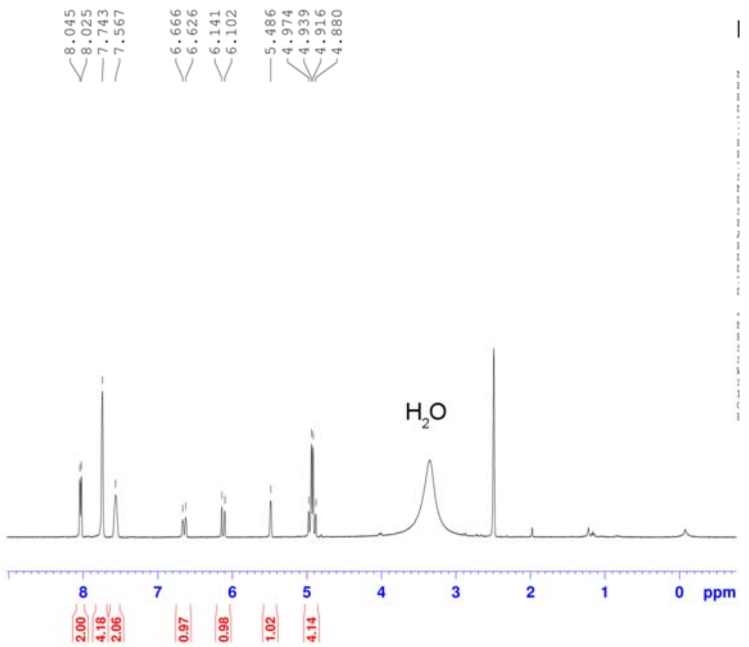

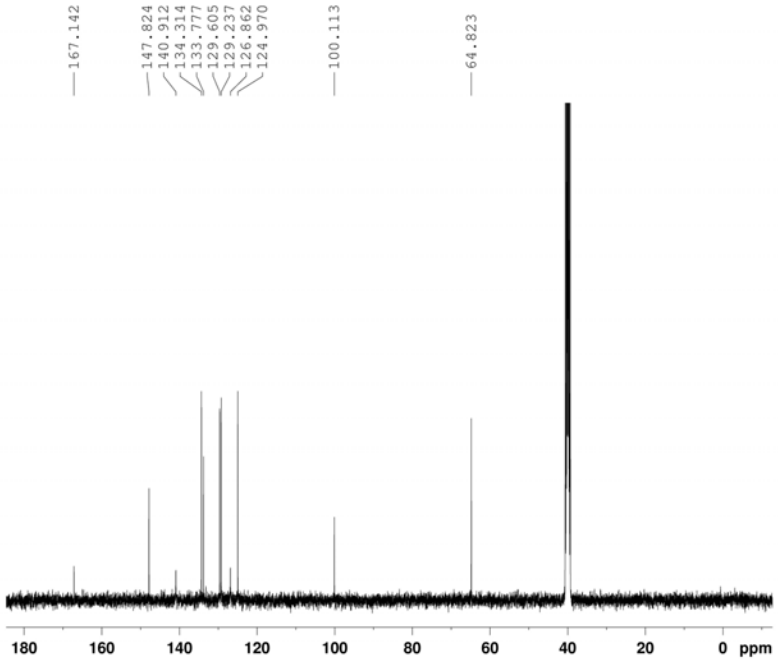

NMR of Compound **4**

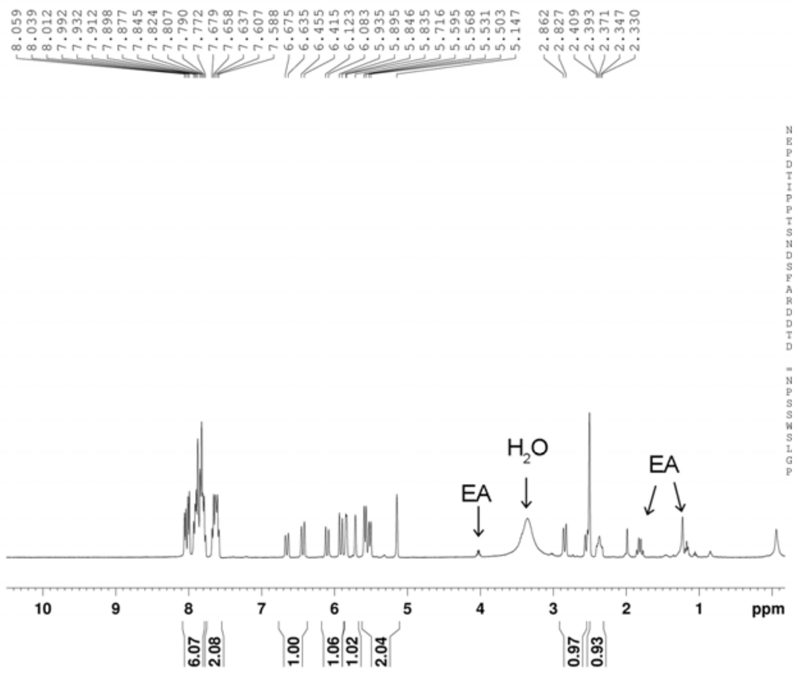

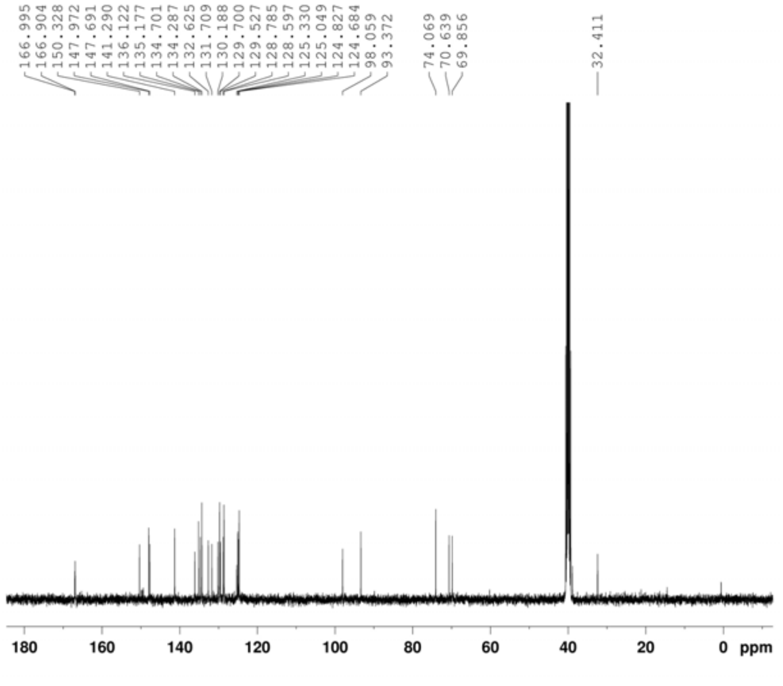

NMR of Compound **5**

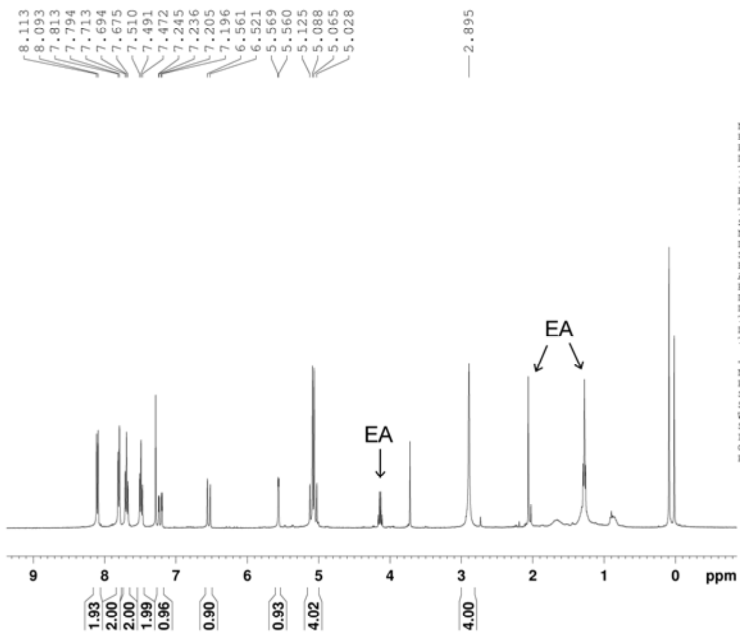

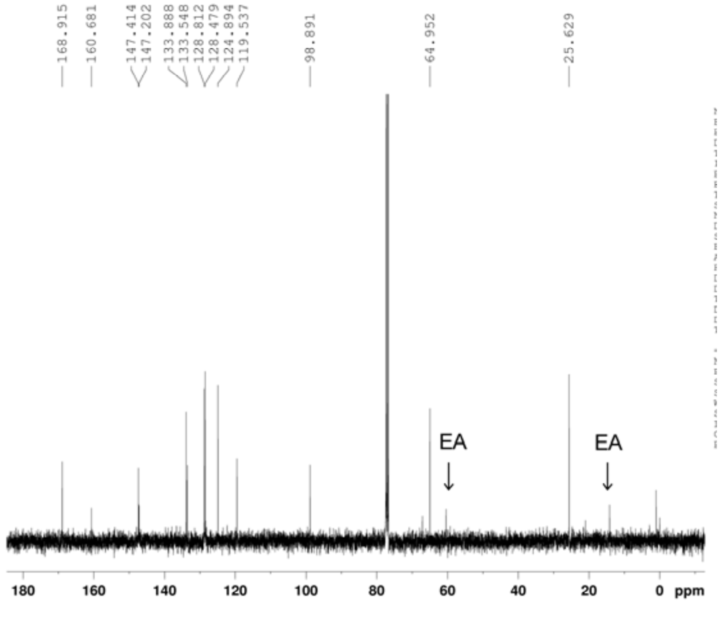

NMR of Compound **6**

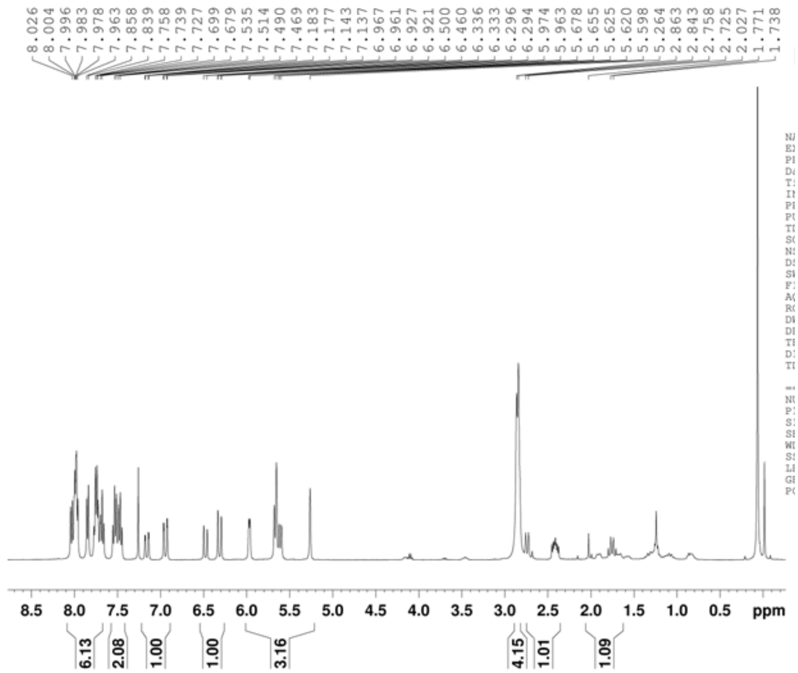

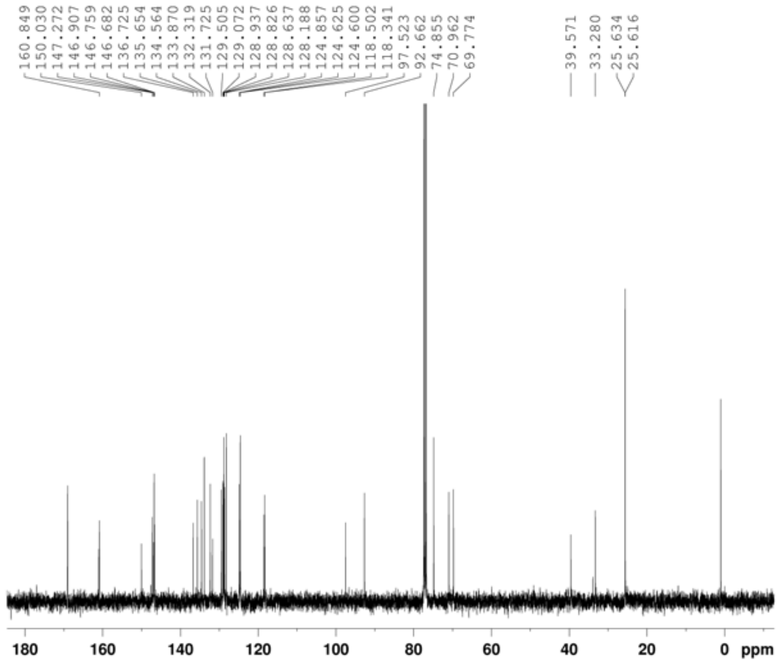

NMR of Compound **7**

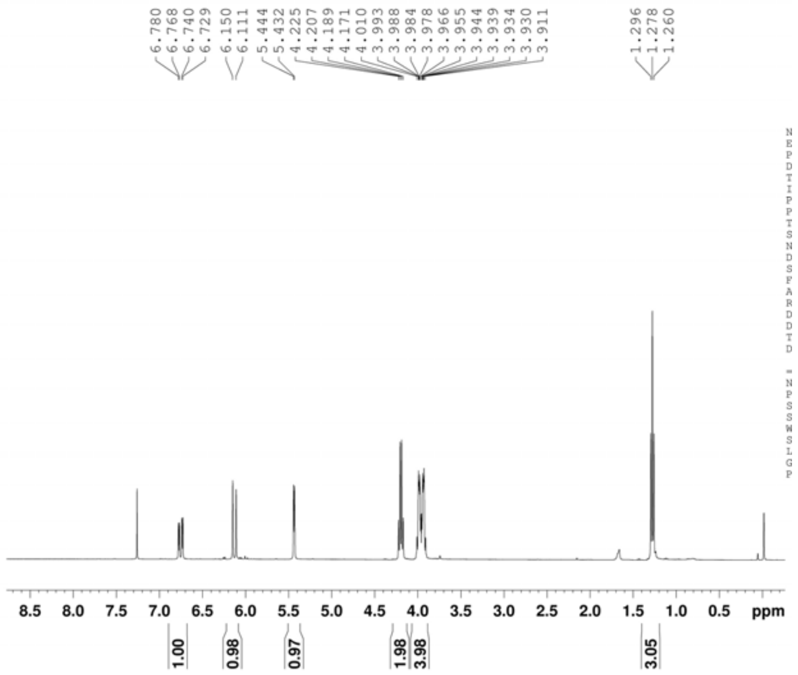

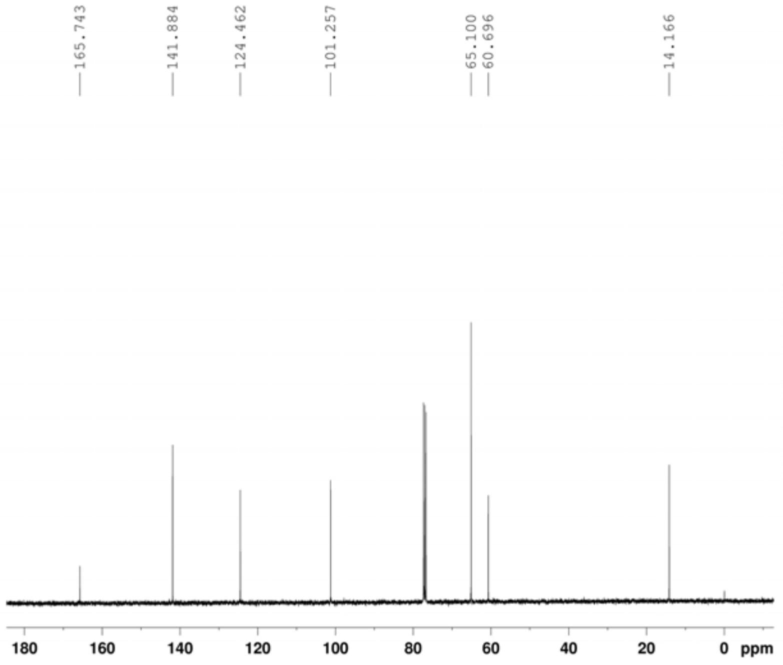

NMR of Compound **8**

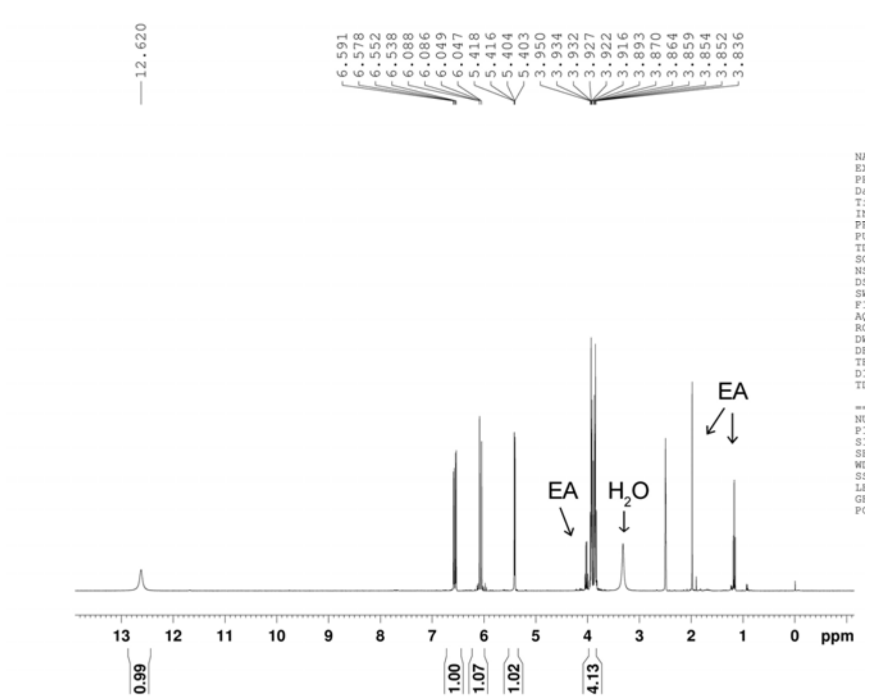

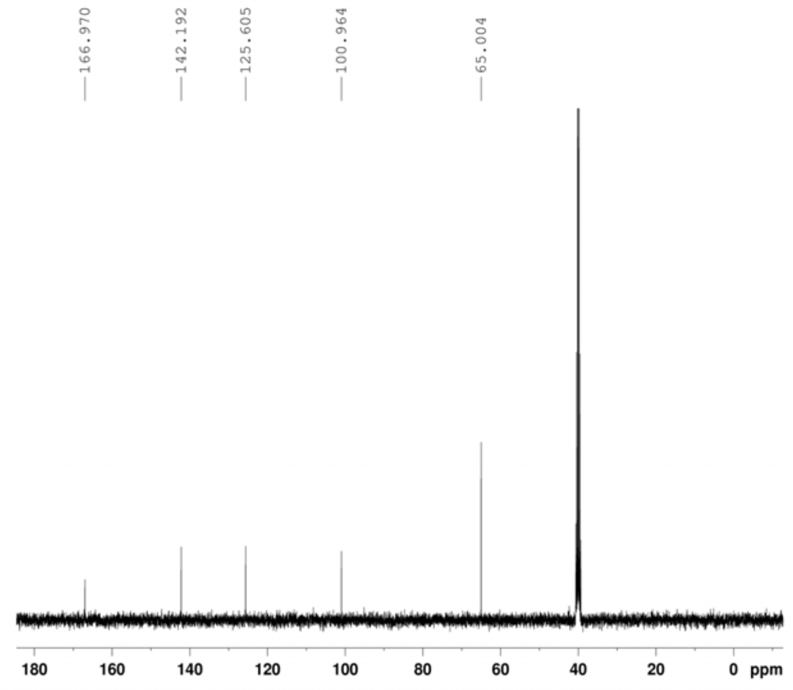

NMR of Compound **9**

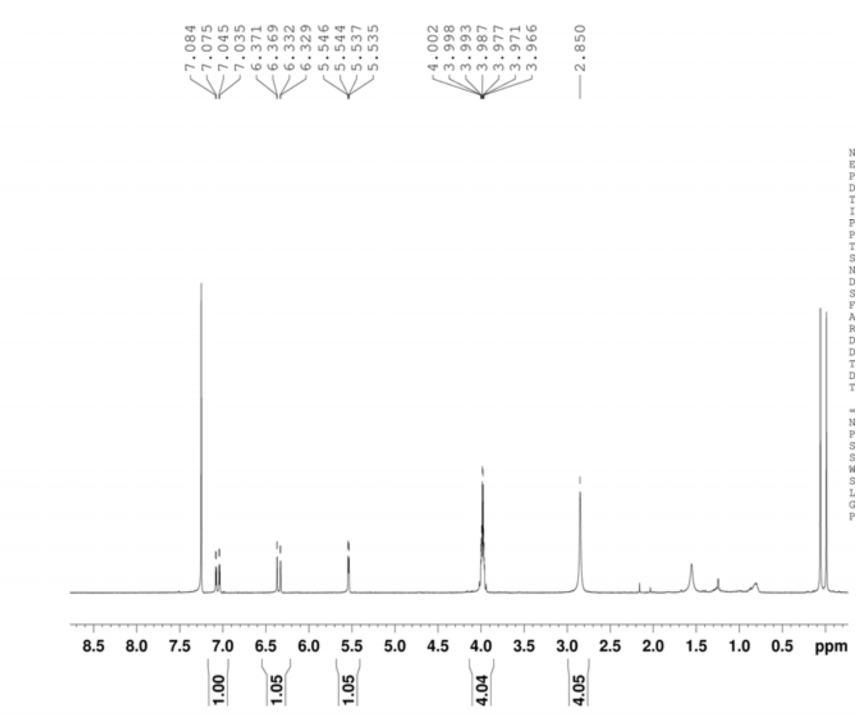

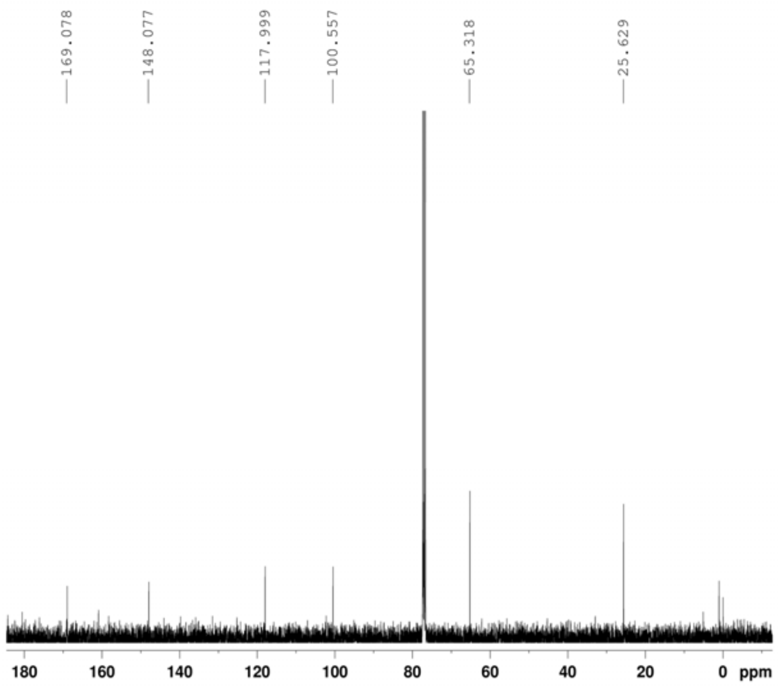

NMR of Compound **10**

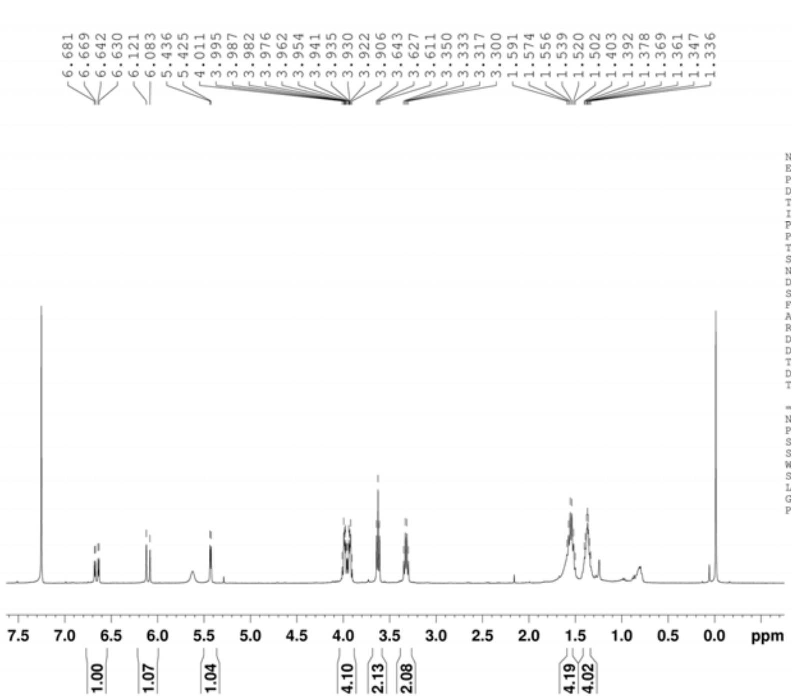

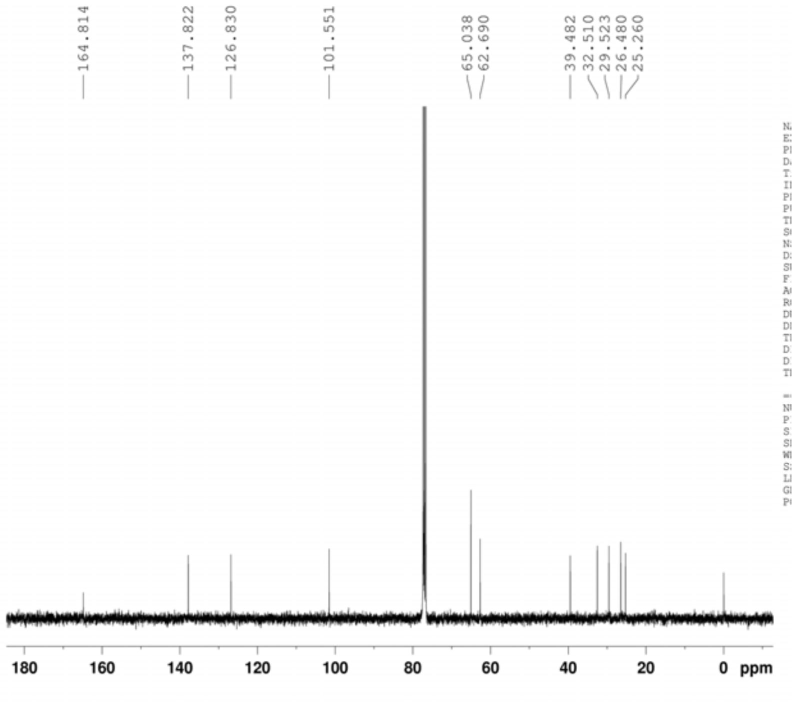

NMR of Compound **11**

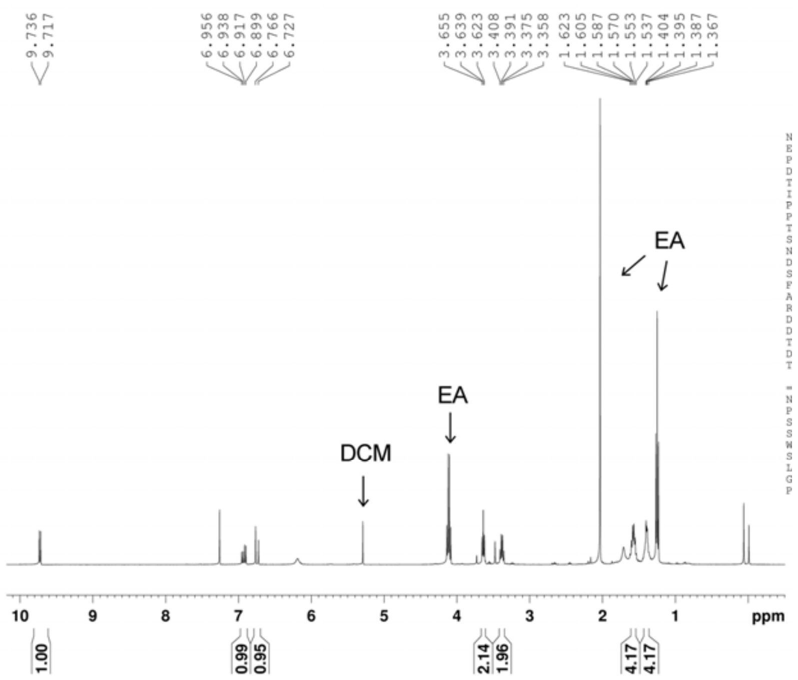

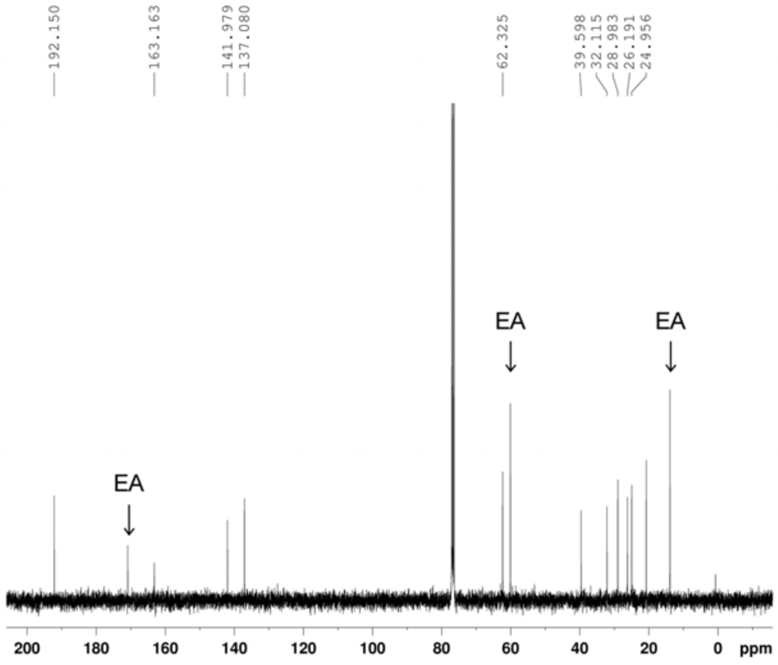

NMR of **P1, 2**

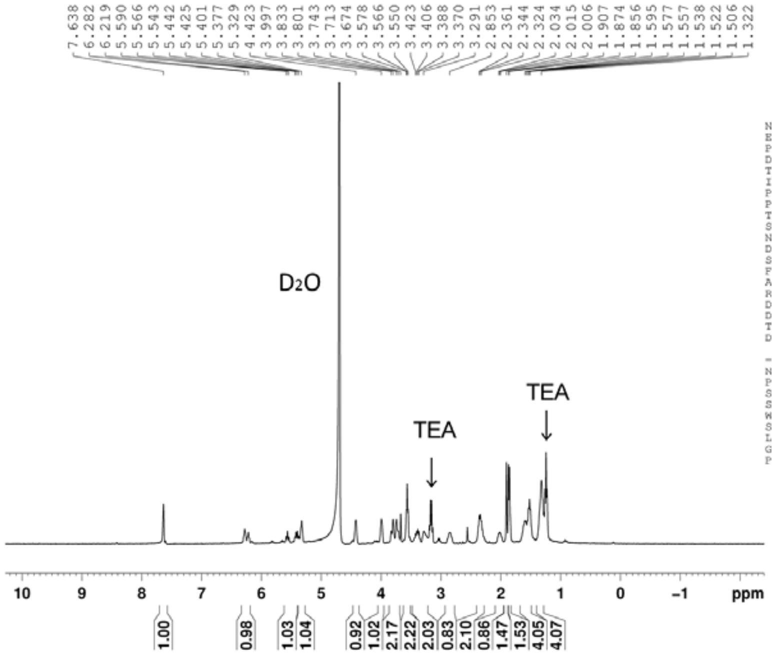
7.638 6.282 6.219 5.590 5.566 5.543 5.442 5.425 5.401 5.377 5.329 4.423 3.997 3.833 3.801 3.743 3.713 3.674 3.578 3.566 3.550 3.423 3.406 3.388 3.370 3.291 2.853 2.361 2.344 2.324 2.034 2.015 2.006 1.907 1.874 1.856 1.595 1.577 1.557 1.538 1.522 1.506 1.322
D2O
TEA
TEA
10 9 8 7 6 5 4 3 2 1 0 −1 ppm
1.00 0.98 1.03 1.04 0.92 1.02 2.17 2.22 2.03 0.83 2.10 0.86 1.47 1.53 4.05 4.07

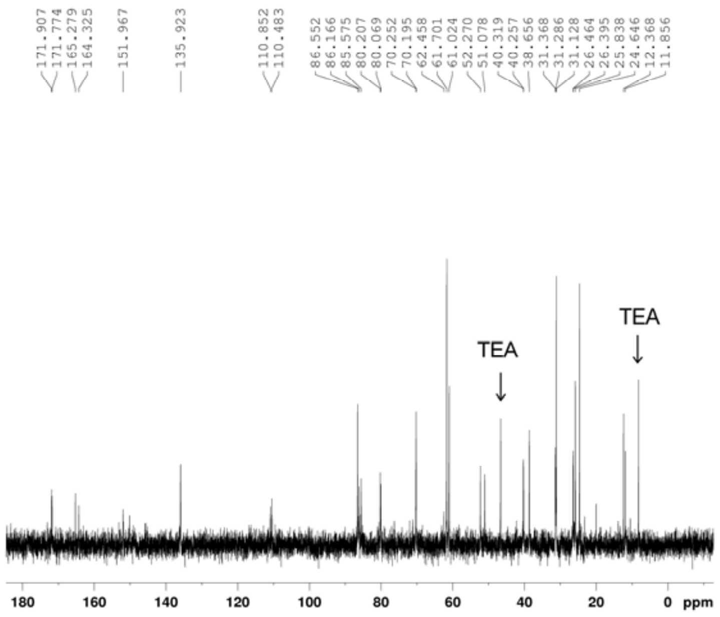
171.907 171.774 165.279 164.325 151.967 135.923 110.852 110.483 86.552 86.166 85.575 80.207 80.069 70.252 70.195 62.458 61.701 61.024 52.270 51.078 40.319 40.257 38.656 31.368 31.286 31.128 26.464 26.395 25.838 24.646 12.368 11.856
TEA
TEA
180 160 140 120 100 80 60 40 20 0 ppm

NMR of Compound **P3**

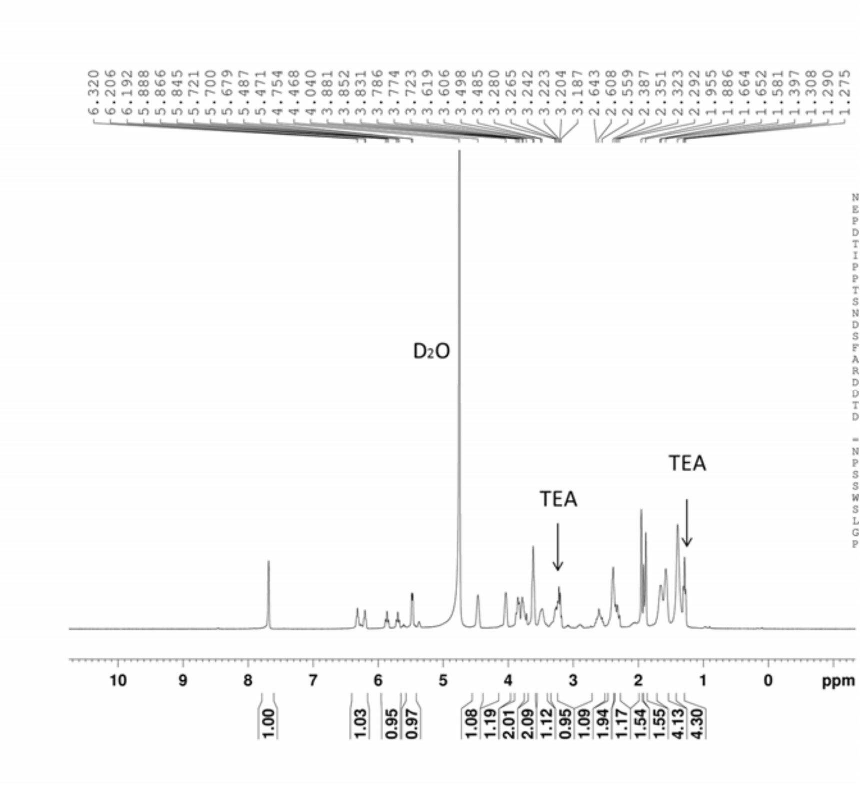

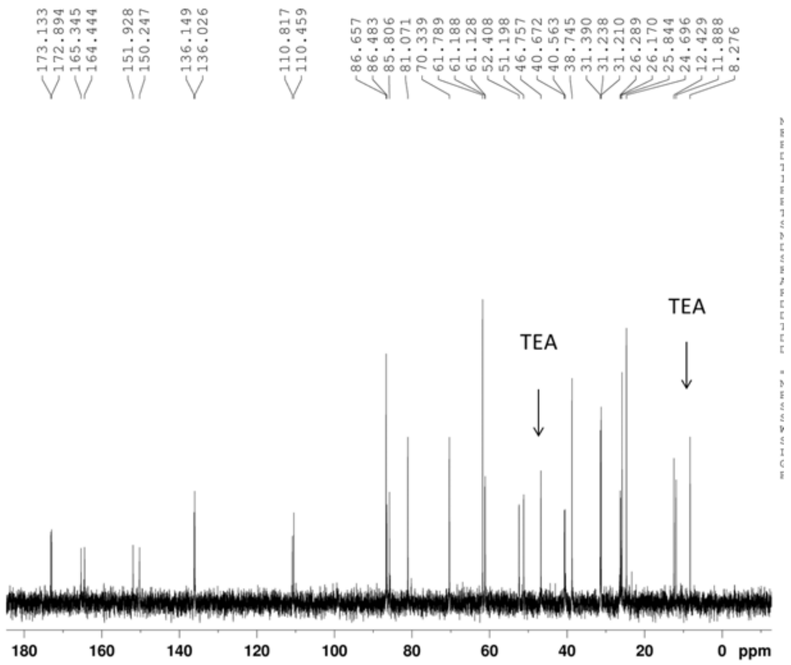

MASS of Compound **5**

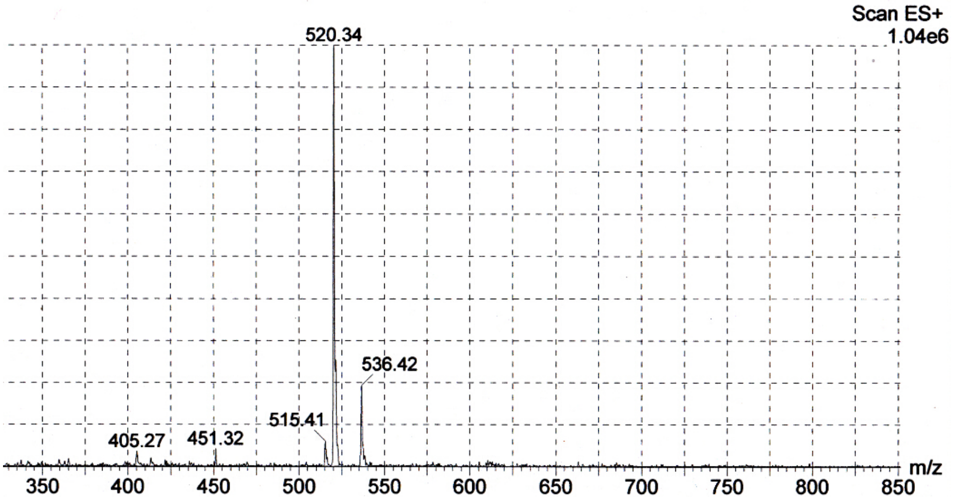

MASS of Compound **6**

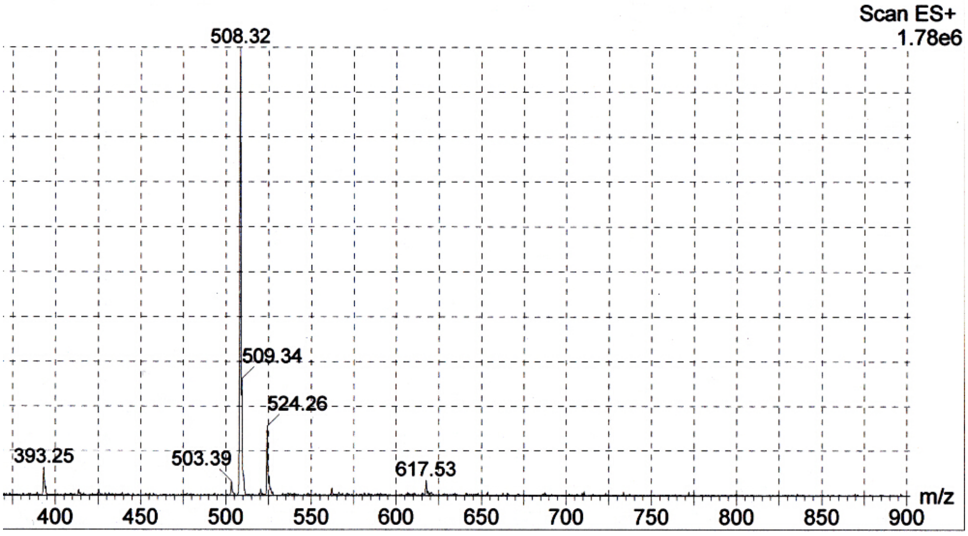

MASS of Compound 11

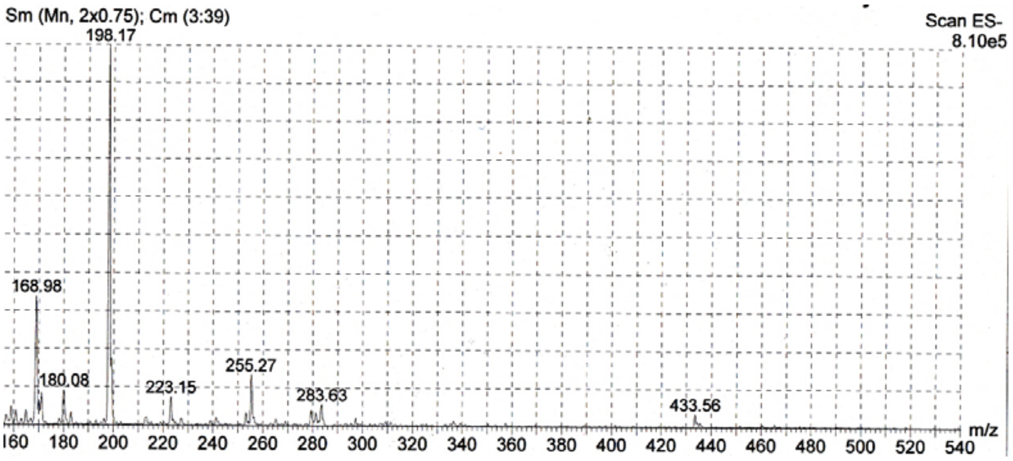

MASS of Compound P1,2

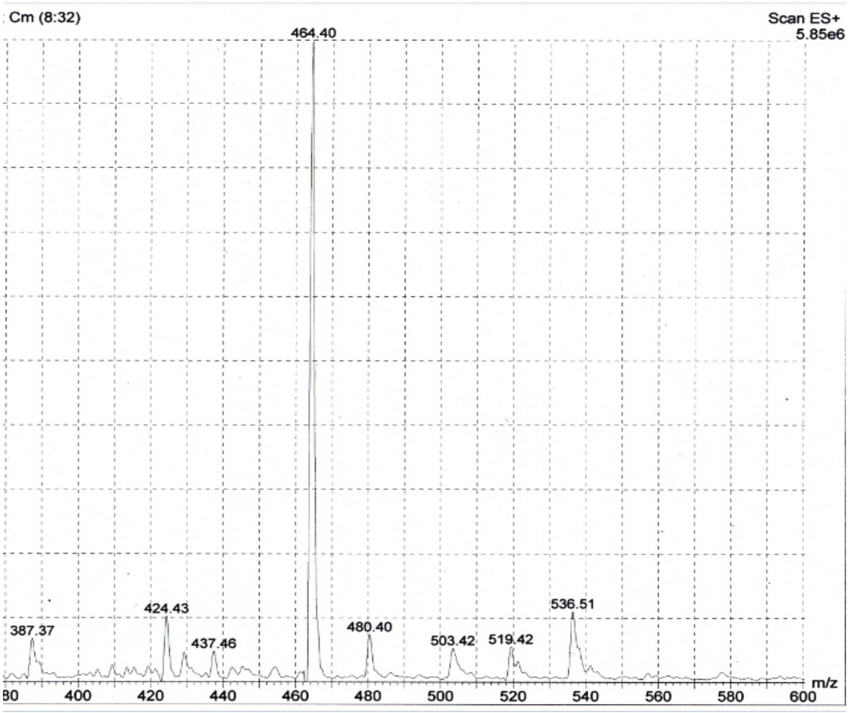

MASS of Compound P3

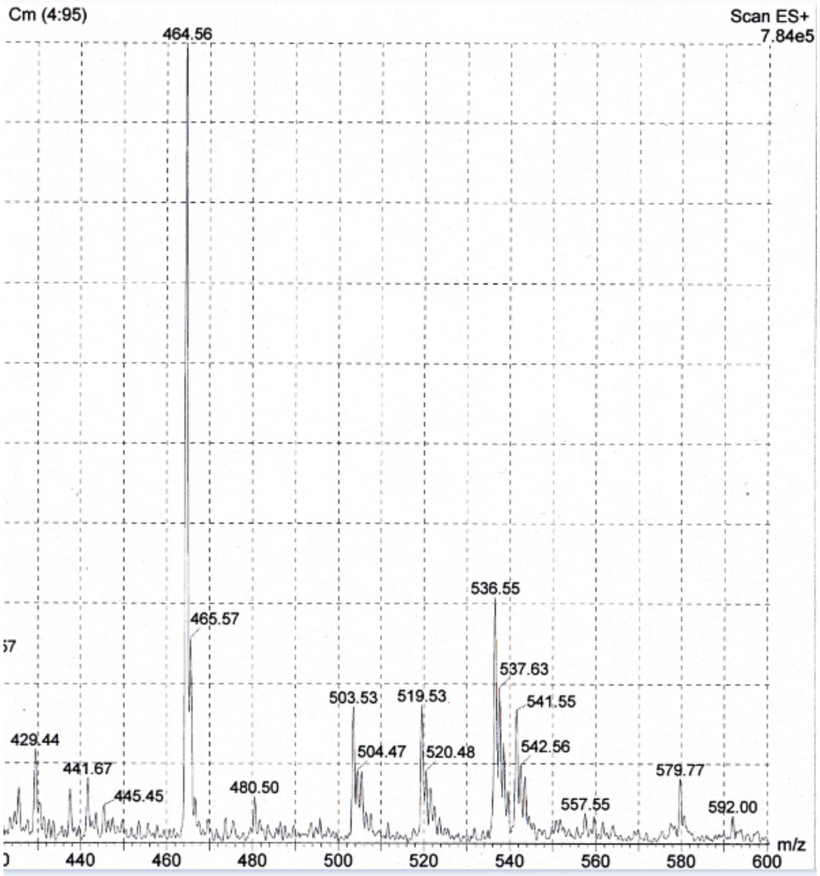

MASS of Enzymatic Hydrolyzates Products

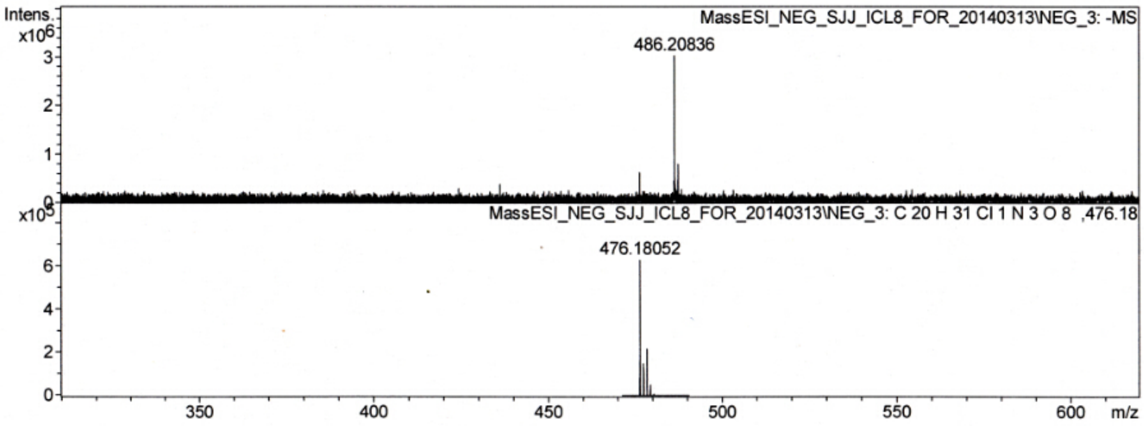

MS of ODN **1**

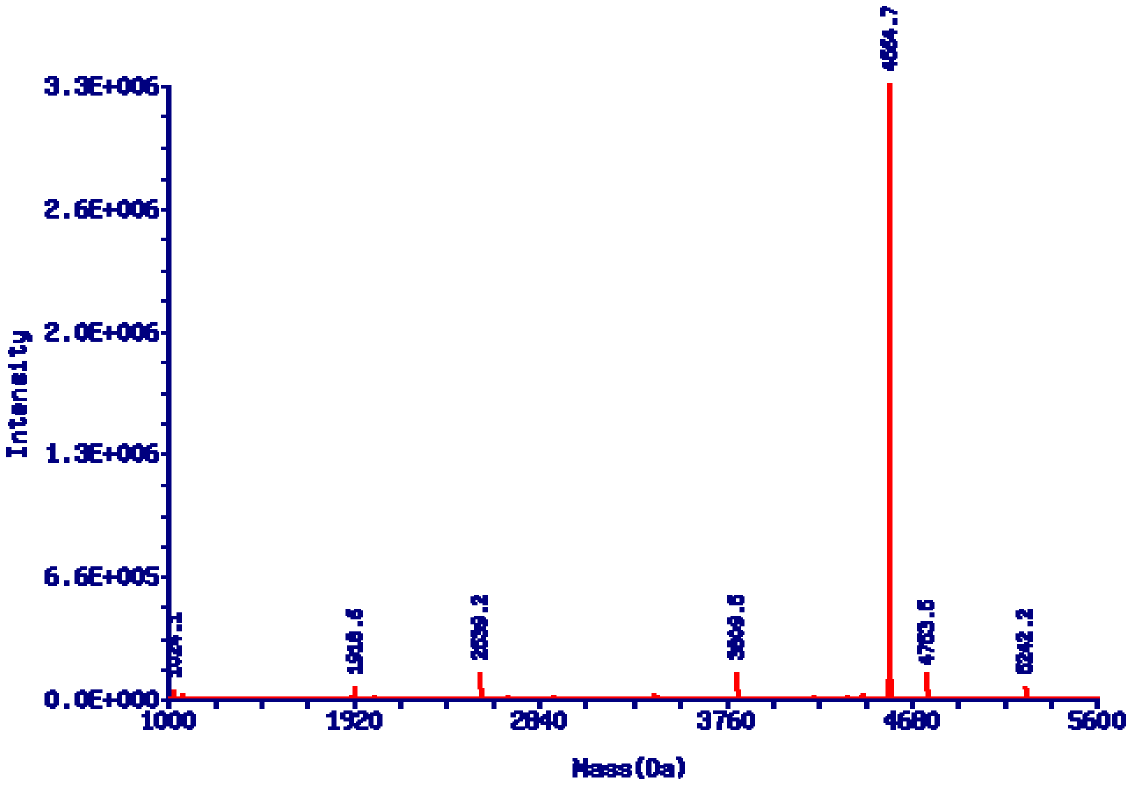

MS of ODN **2**

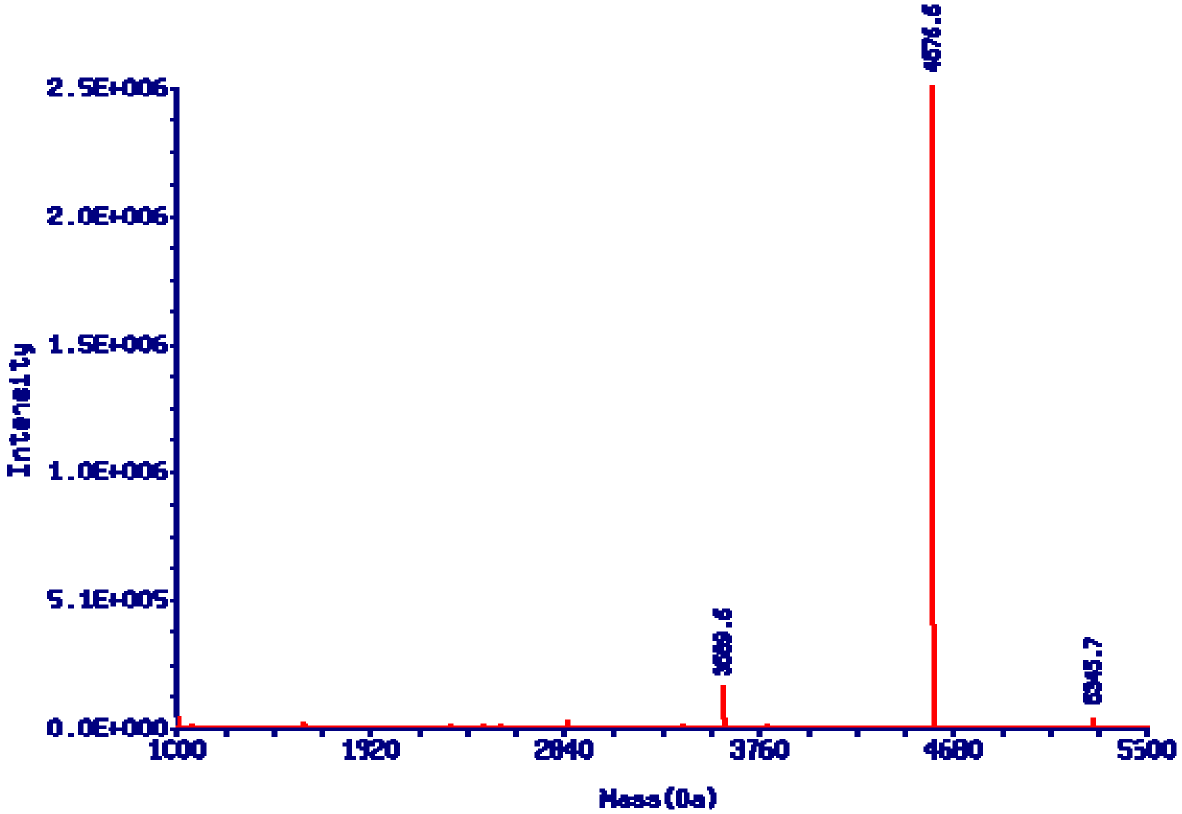

MS of ODN **3**

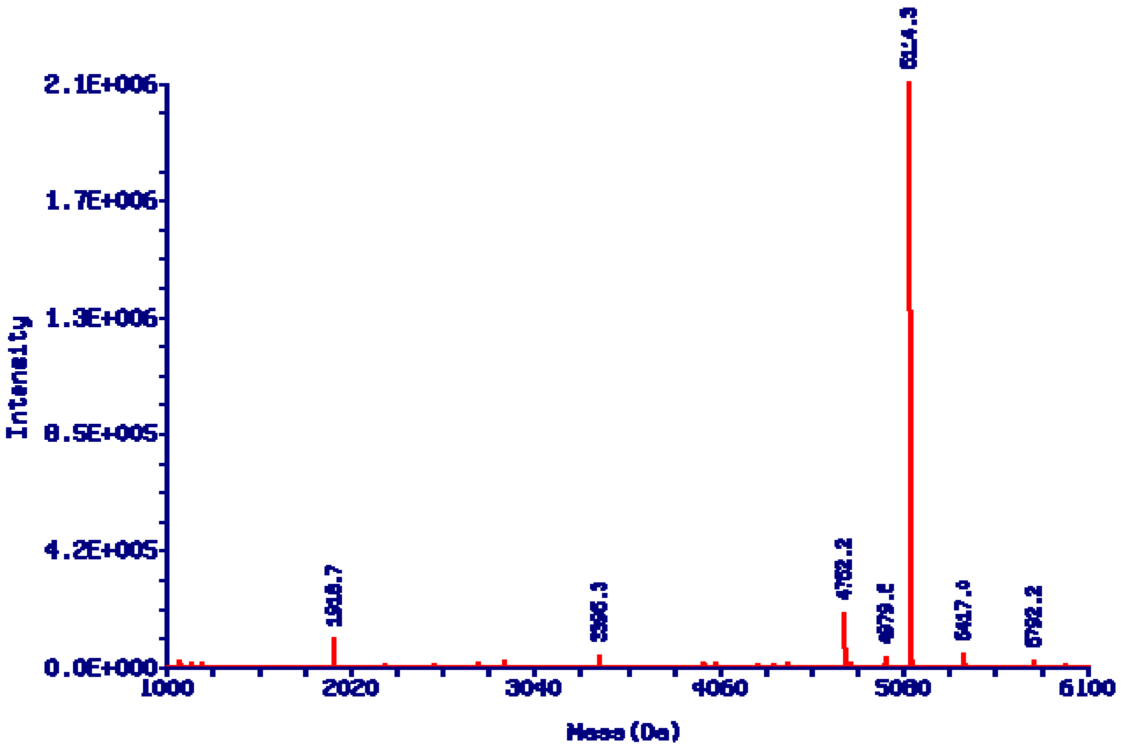

Supplement: Supplementary Information [file srep10473-s1.pdf]
